# High Ionic Seebeck Effect in Natural Leaves

**DOI:** 10.1002/adma.202510413

**Published:** 2025-07-26

**Authors:** Hungu Kang, Hongwoo Lee, Cheljong Hong, Jiung Jang, Sahar Ayachi, Xin He, Pil Joon Seo, Alois Würger, Hyo Jae Yoon

**Affiliations:** ^1^ Department of Chemistry Korea University Seoul 02841 South Korea; ^2^ Department of Chemistry Seoul University Seoul 08826 South Korea; ^3^ Université de Bordeaux and CNRS LOMA (UMR 5798) Talence 33405 France

**Keywords:** ionic seebeck, ions, leaf, thermovoltage

## Abstract

Plants actively transport ions to sustain life, yet their capacity to convert heat to electricity via the ionic Seebeck effect has remained unexplored. Here, it is demonstrated that natural *Ficus elastica* leaves can generate ionic thermovoltages up to 7 V under mild temperature gradients, achieving a high ionic figure of merit of ∼5.6 at room temperature. This pronounced thermoelectric response arises from anion thermodiffusion through the apoplast and is significantly amplified by leaf desiccation and electrode selection. A dielectric capacitive model accounts for the observed enhancement. Notably, living leaves generate thermopower under light‐induced temperature gradients, underscoring the potential of in vivo ionic thermoelectric assays. These findings reveal an unrecognized energy‐harvesting function in plant tissue and introduce a biodegradable platform for sustainable thermal‐to‐electrical energy conversion.

## Graphical abstract

This work reveals a role of natural leaves as thermoelectric biomaterials, capable of converting heat into electricity through the ionic Seebeck effect. The leaves exhibit significant and reproducible thermopower under both moderate temperature gradients and photothermal conditions. These findings open avenues for developing eco‐friendly energy conversion systems and biotechnologies based on in vivo thermoelectric assay.

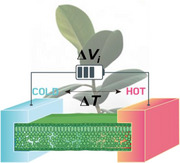

## 1 Introduction

The ionic Seebeck effect^[^ ^1^, ^2^, ^3^ ^]^ is a thermally driven transport phenomenon in which ions migrate at different speeds depending on their mass, size, activation energy, and Coulombic interactions.^[^ ^4^ ^]^ When a temperature differential (Δ*T*, K) is applied across an electrolyte, ions accumulate asymmetrically at the two electrodes, forming electric double layers and generating a measurable ionic thermovoltage (Δ*V*_i_, mV).^[^ ^1^, ^2^, ^3^ ^]^ The resulting ionic Seebeck coefficient (*S*_i_, mV K^−1^) is defined as Δ*V*_i_ / Δ*T* and is governed by the heat transport *Q_i_*, the ionic valences *z_i_* (±1,±2,…), and concentration *n_i_* of the mobile charge carriers:^[^ ^5^, ^6^ ^]^

(1) $$\;S_{i}=\frac{1}{eT}\frac{\sum_{i}z_{i}n_{i}Q_{i}}{\sum_{i}z_{i}^{2}n_{i}}$$

This effect has attracted growing interest as a mechanism for energy harvesting in soft‐matter systems. The Seebeck coefficient in synthetic materials—such as ionic gels and organic polymers—has typically remained in the range of tens of millivolts per kelvin.^[^ ^7^, ^8^ ^]^

In plants, ion transport underlies a range of biological functions, including nutrient uptake,^[^ ^9^ ^]^ osmo‐ and pH‐regulations of cellular environments,^[^ ^10^ ^]^ energy production,^[^ ^11^ ^]^ and stress response.^[^ ^12^ ^]^ Leaf tissues are rich in natural polymers such as pectin and (hemi)cellulose, which form hydrated networks that guide the ion movement. Despite their abundance, the role of these matrices—especially pectin—in regulating thermodiffusion has been largely overlooked.

Here, we investigate the ionic Seebeck effect in *Ficus elastica* leaves using a thermodiffusion cell that measures Δ*V*_i_ as a function of Δ*T*. We find that thermopower is primarily governed by anion transport through the apoplast. While fresh leaves show moderate Seebeck coefficients (on the order of several mV K^−1^), these values increase dramatically upon desiccation. Both polarity and magnitude of *S*_i_ are highly sensitive to the choice of electrode material. Using carbon tape electrodes on desiccated leaves, we achieved *S*_i_ values as high as 334 mV K^−1^ and an ionic figure of merit (*ZT*) of ≈5.6 at room temperature (298 K). Extended drying for four days amplified the thermoelectric response, yielding an *S*_i_ of 971 mV K^−1^ and a thermovoltage approaching 7 V under a Δ*T* of just 10 K—representing an unprecedented performance in natural systems. To explain this enhancement, we propose a dielectric capacitive model in which desiccation forms a low‐permittivity surface layer that generates an interfacial polarization field augmenting the thermovoltage. The model is supported by synthetic paper‐based systems with dielectric layers. Finally, we demonstrate a non‐destructive, in vivo ionic thermoelectric system using living leaves, establishing a biodegradable platform for sustainable ionic thermal‐to‐electric energy applications.

## 2 Results and Discussion

### 2.1 Structure of Leaf

**Figure 1:**
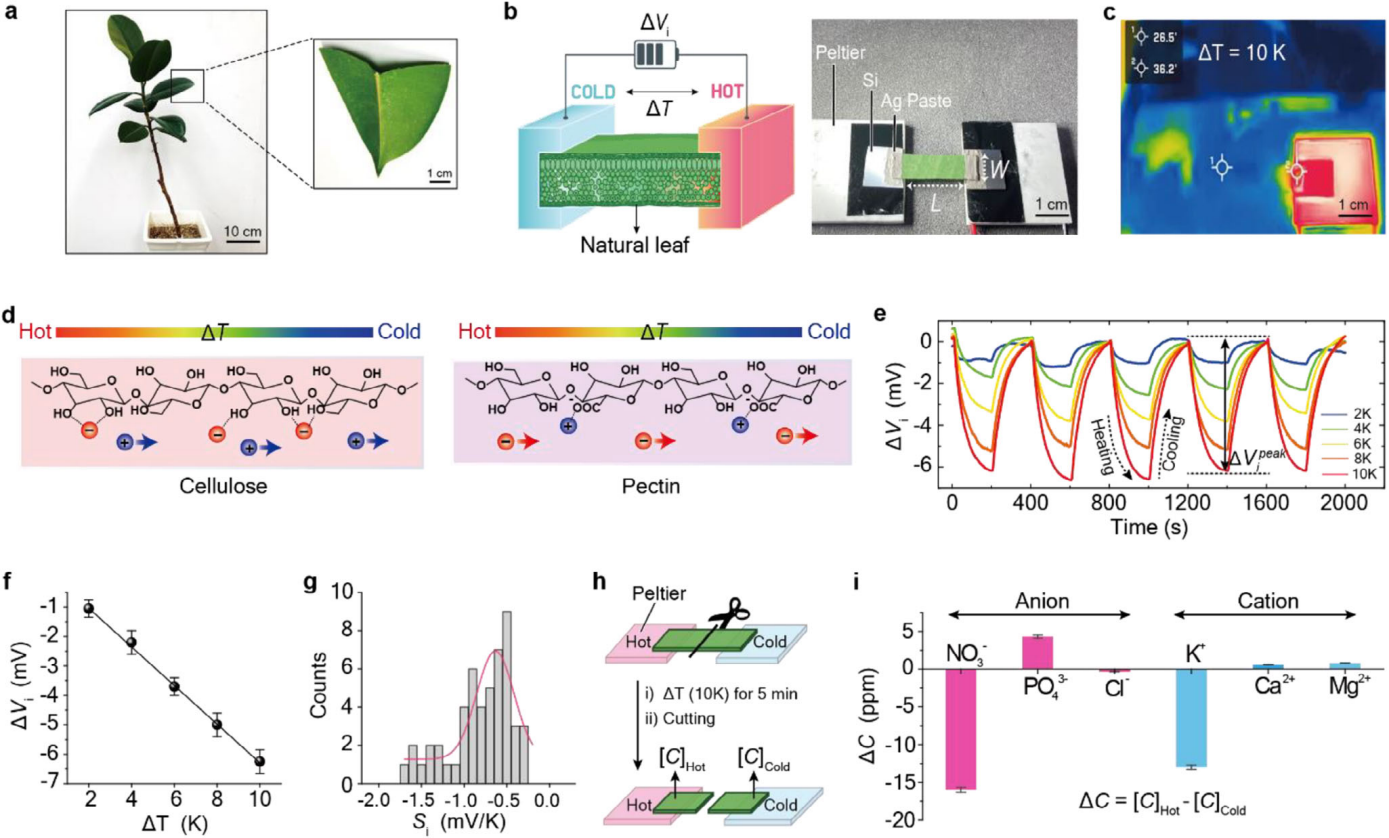
Experimental setup and ionic thermovoltage analysis. a) Photographs of a *Ficus elastica* leaf used the experiments. b) Left: Schematic of the leaf‐based thermodiffusion measurement setup. Right: Photograph of the assembled electrode‐leaf‐electrode thermodiffusion cell (leaf dimensions: width (*W*) = 1 cm and length (*L*) = 2 cm). c) Thermographic image showing the temperature gradient (*∆T*) applied to the leaf using a Peltier device. d) Schematic illustrating ion thermodiffusion in cellulose and pectin matrices under a temperature gradient. Cellulose facilitates cation diffusion, while its hydroxyl groups interact with anions (left); pectin promotes anion diffusion via carboxyl (COO^−^) group interactions with cations (right). e) Ionic thermovoltage (*∆V*_i_) curves recorded over five heating/cooling cycles for a fresh leaf‐based cell. *∆* $V_{i}^{peak}$ denotes the peak voltage at each *∆T*. f) Plot of *∆V*_i_ versus *∆T*, showing a linear relationship. Error bars indicate the standard deviation measured from five heating/cooling cycles. g) Histogram of ionic Seebeck coefficients (*S*_i_, mV K^−1^) obtained from 50 separate leaf‐based thermodiffusion cells. h) Schematic of the sample preparation for ion chromatography analysis, involving leaf segmentation after applying a temperature gradient. i) Ion concentration differences (Δ*C*_cold‐hot_) between the cold and hot regions of the leaf, determined by ion chromatography. Error bars represent the standard deviation from three independent ion chromatography measurements of Δ*C*_cold‐hot_.

In this study, we used *Ficus elastica* leaves, commonly known as Indian rubber tree (**Figure** **1**a), as a proof‐of‐concept platform for studying the ionic Seebeck effect. The robustness of these leaves makes them ideal for investigating the ionic Seebeck effect. A typical leaf consists of mesophyll layers enclosed by upper and lower epidermis (Figure S1, Supporting Information). At the molecular level, the plant cell wall is primarily composed of cellulose (≈30 %, w w^−1^), hemicellulose (≈30 %), lignin (≈5 %), and pectin (≈35 %), which collectively adsorb and retain water and various ions (Figure S2a, Supporting Information).^[^ ^13^, ^14^ ^]^ To visualize the presence of cellulose and pectin in the cell walls of leaves we used, we employed staining methods specific to each component. Leaves were submerged in 0.04% Congo Red and 0.04% Ruthenium Red solutions for 30 min to stain cellulose and pectin, respectively.^[^ ^15^, ^16^ ^]^ After thorough rinsing, stained upper epidermal layers were observed under a microscope (Figure S2b, Supporting Information). To further confirm the presence of these polymers, we performed chemical extraction using established protocols (Figure S3, Supporting Information).^[^ ^17^, ^18^ ^]^

### 2.2 Ionic Seebeck Effect of Fresh Leaf

To investigate the ionic Seebeck effect in leaves, we cut a leaf into a 1 cm × 2 cm rectangle and constructed a cell by applying Ag paste electrodes to both ends (Figure 1b). The leaf was≈300 µm thick. A temperature gradient (*∆T*) was applied by heating one end with a Peltier device while maintaining the other at ambient temperature, as confirmed by thermographic imaging (Figure 1c). *∆T* was varied from 2 to 10 K, with each heating step lasting 200 s. The Peltier temperature showed a linear correlation with the measured *∆T* (Figure S4, Supporting Information).

The leaf matrix is composed primarily of cellulose, hemicellulose, and pectin, each contributing distinct ion‐binding functionalities. Cellulose and hemicellulose contain hydroxyl (–OH) groups, which interact with anions via hydrogen bonding, thereby enhancing cation thermodiffusion.^[^ ^8^ ^]^ In contrast, pectin contains both hydroxyl and carboxylate (−COO^−^) groups, which strongly bind cations and promote anion diffusion (Figure 1d). The competition between the opposing effects can be probed using fresh leaf‐based thermodiffusion cells. *∆V*_i_ was recorded over synchronous heating and cooling cycles at each Δ*T* and processed using the baseline correction method described in Figure S6 (Figure 1e). The *S*_i_ was estimated from the slope of the *∆V*_i_ versus *∆T* plot.^[^ ^4^, ^19^, ^20^ ^]^ Figure 1f illustrates the peak *∆V*_i_ (*∆* $V_{i}^{peak}$) values against *∆T* for a freshly prepared leaf. Measurements from 50 separate samples confirmed that the fresh leaves exhibit a reproducible *S*_i_ value of −0.64 ± 0.22 mV K^−1^ (Figure 1g), indicating anion‐dominant transport. The *S*_i_ value was independent of leaf length (i.e., electrode‐to‐electrode distance; Figures S7 and S8, Supporting Information).

### 2.3 Identifying Ion Species

To determine the primary ion species contributing to the ionic Seebeck effect in leaves, we applied a temperature gradient of 10 K for 5 min. After exposure, the leaf was segmented into hot and cold regions for ion chromatography analysis (Figure 1h). Leaves generally contain various ionic species, among which K^+^, Mg^2+^, Ca^2+^, NO_3_^−^, and PO_4_^3−^ are typically dominant,^[^ ^21^ ^]^ leading us to focus on these ions. The concentrations of NO_3_^−^, PO_4_^3−^, and K^+^ ions exhibited significant differences between the hot and cold regions, whereas Cl^−^, Ca^2+^, and Mg^2+^ levels remained relatively similar (Figure 1i; Figures S9–S12, Supporting Information). Notably, PO_4_^3−^ accumulated more in the cold region, while NO_3_^−^ was more concentrated in the hot region. Given that PO_4_^3−^ carries a higher negative charge than NO_3_^−^, its thermodiffusion likely plays a crucial role in the observed negative ionic Seebeck coefficient in fresh leaves. Furthermore, the lower concentration of K^+^ on the cold side further supports the anion‐dominant transport and the observed negative *S*_i_ value. It is noteworthy that other ion species such as F⁻, NO₂⁻, Br⁻, SO₄^2^⁻, I⁻, and Li⁺ were not detected in our analysis while NH₄⁺ and Na⁺ were detected but showed no significant concentration difference between the hot and cold sides, indicating the absence of thermally driven ionic transport for these ions.

### 2.4 Ion Transport Pathway

**Figure 2:**
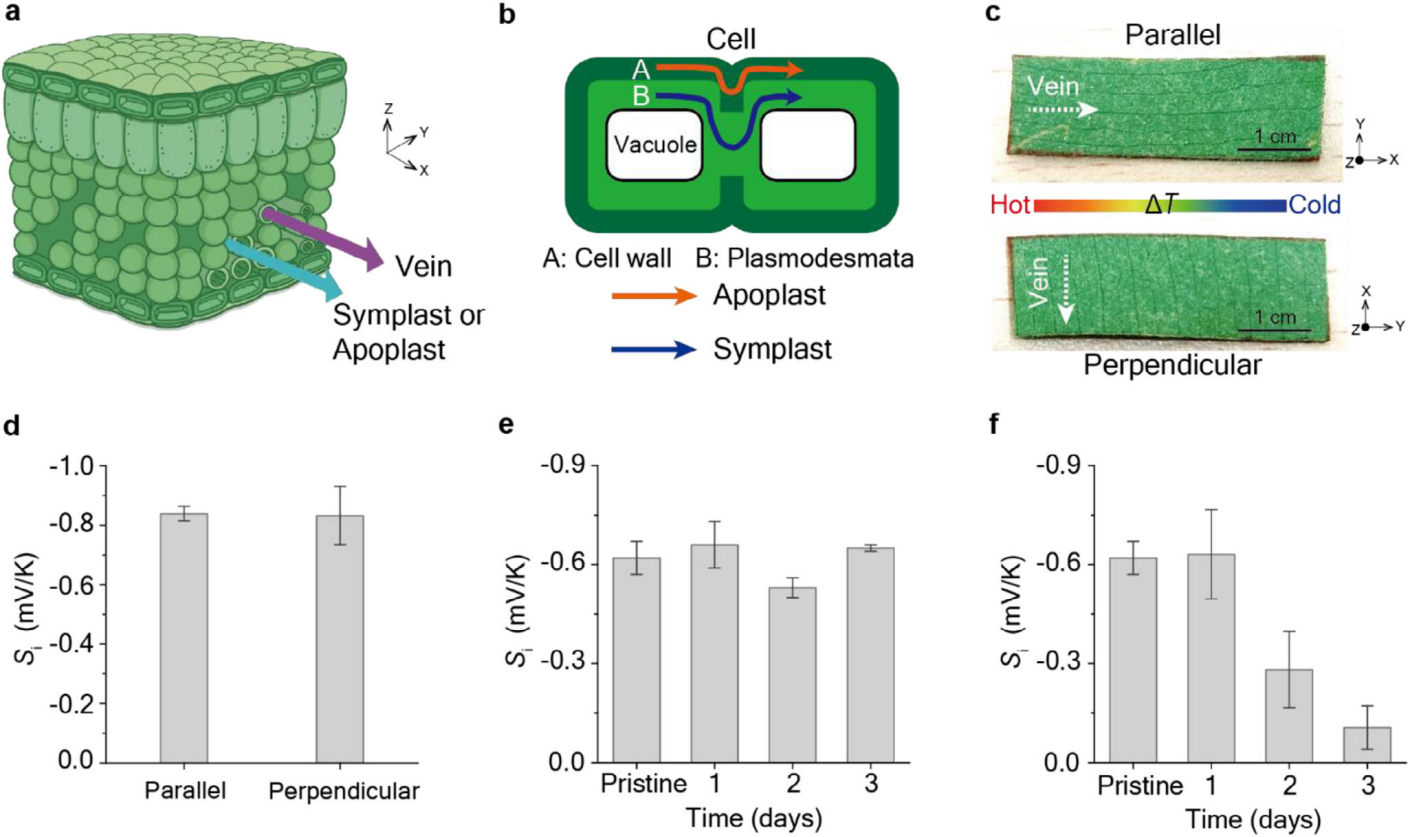
Ion transport pathways in leaves and their influence on the ionic Seebeck effect. a) Schematic of leaf anatomy showing ion transport via the vein, symplast, and apoplast pathways. b) Schematic of two ion transport pathways in plant tissue: the apoplastic pathway (via the cell wall) and the symplastic pathway (via plasmodesmata). c) Photographs depicting the orientation of leaf veins relative to the applied temperature gradient (*∆T*), with samples prepared in either parallel or perpendicular alignments. d) Comparison of ionic Seebeck coefficient (*S*_i_) values for leaves with veins aligned parallel or perpendicular to the *∆T* direction, showing no significant difference. e) Leaves were treated with cycloheximide (CHX) as the symplast inhibitor for 1–3 days, showing minimal impact on *S*_i_. f) Leaves treated with CuSO_4_/K_4_[Fe(CN)_6_] as the apoplast inhibitor for 1–3 days showed a marked decrease in *S*_i_ over time. Error bars in (d–f) indicate the standard deviation calculated from three independent measurements for each data point.

Within a leaf, ions can traverse three main pathways: i) apoplast, ii) symplast, iii) vein (xylem) (**Figure** **2**a). The symplastic pathway facilitates cell‐to‐cell molecular transport through plasmodesmata, and the apoplastic pathway enables passive, non‐selective transport through cell walls, primarily composed of pectin and cellulose (Figure 2b).^[^ ^22^ ^]^ If the veins were the dominant route, the ionic Seebeck effect would depend on the orientation of the leaf sample, due to the vein's inherent directionality (Figure 2c; Figures S17 and S18, Supporting Information). However, measurements revealed that *S*_i_ values were nearly identical when the veins were aligned either parallel (‐0.84 ± 0.02 mV K^−1^) or perpendicular (−0.83 ± 0.10 mV K^−1^) to the *∆T* direction (Figure 2d). This indicates that the ionic Seebeck effect is not dominated by ion transport through the veins.

To determine whether the symplast or apoplast governs the ionic Seebeck effect in leaves, we selectively inhibited each pathway using pathway‐specific chemical treatments. Cycloheximide (CHX), a well‐established symplast inhibitor^[^ ^23^ ^]^ was applied at a concentration of 0.05% (v/v) in an aqueous DMSO solution for one to three days. The *S_i_* of CHX‐treated leaves remained unchanged over incubation time and were comparable to those of untreated control (Figure 2e; Figures S19 and S20, Supporting Information). In contrast, leaves treated with an apoplast inhibitor—formed by the in situ precipitation of CuSO_4_/K_4_[Fe(CN)_6_] in aqueous solution^[^ ^24^, ^25^ ^]^—exhibited a time‐dependent decrease in *S_i_*, from −0.63 ± 0.14 to ‐0.11 ± 0.07 mV K^−1^ (Figure 2f; Figures S19 and S21, Supporting Information). These results indicate that the ionic Seebeck effect in leaves is primarily governed by ion transport through the apoplast rather than the symplast.

### 2.5 Desiccation Effects

**Figure 3:**
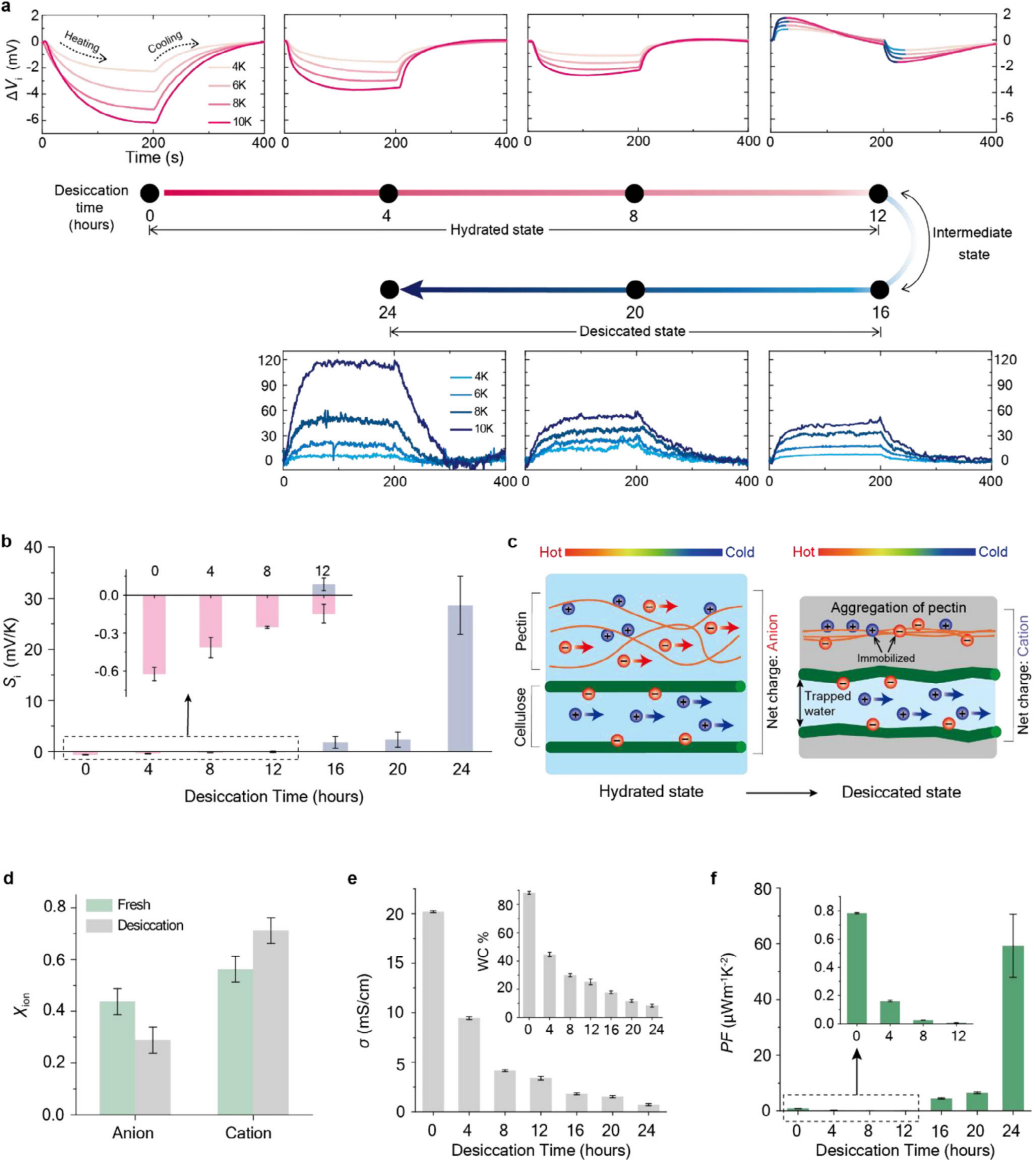
Effect of desiccation on ionic thermovoltage and material properties of leaves. a) Ionic thermovoltage (Δ*V*) curves recorded at 4‐h desiccation intervals. Heating was applied for the first 200 s, followed by cooling for the next 200 s. Color‐coded lines indicate ion type: pink for anion thermodiffusion and blue for cation thermodiffusion. b) Plot of the ionic Seebeck coefficient (*S*_i_, mV K^−1^) as a function of desiccation time. c) Schematic illustrating structural changes in pectin chains and cellulose microfibrils during desiccation, along with corresponding differences in ion thermodiffusion. Ion transport through pectin is significantly hindered by drying, whereas transport through cellulose is minimally affected. d) Ion mole fraction (χ_ion_) of anions and cations in the cold region of fresh and 24‐h desiccated leaves, determined by ion chromatography. e) Ion conductivity (*σ*, mS cm^−1^) as a function of desiccation times, showing a gradual decrease. f) Power factor (PF, µWm^−1^K^−2^) versus desiccation time, revealing an initial drop followed by a marked increase after 12 h. Error bars in (b, d–f) indicate the standard deviation obtained from three independent measurements for each data point.

To investigate how desiccation influences on the ionic Seebeck effect in leaves, we cut the leaves into 2 cm × 1 cm sections and gradually desiccated them under ambient conditions and recorded measurements every 4 h. Thermodiffusion cell measurements revealed that the magnitude of negative Δ*V*_i_ progressively decreased over the first 12 h of drying (**Figure** **3**a). This corresponded to a steady decrease in the absolute value of *S*_i_, while its sign remained negative (inset in Figure 3b), indicating continued anion‐dominant thermodiffusion. Between 12 and 16 h of desiccation, a transition in charge carrier polarity, indicative of an intermediate state, was observed: during heating, a positive Δ*V*_i_ initially appeared before decreasing; the reverse pattern occurred during cooling (blue and red lines in the 12‐h desiccation plot in Figure 3a). This transition of polarity suggests an in situ shift from anion to cation dominance. Beyond this point, only positive Δ*V*_i_ values were observed, increasing with both temperature and drying time. Accordingly, the *S*_i_ polarity reversed from negative to positive, reaching a maximum of +28.7 ± 5.7 mV K^−1^ after 24 h of desiccation (Figure 3b). This polarity inversion indicates the dominant ion transport channel changes from pectin to (hemi)cellulose—see below for detailed discussion on this. To further validate the role of desiccation, we sealed leaf samples with Scotch tape to inhibit moisture loss. This significantly slowed the drying process, and the *S*_i_ value changed only slightly while remaining negative—from ‐0.66 ± 0.01 to −0.24 ± 0.02 mV K^−1^ after 24 h (Figures S22 and S23, Supporting Information).

Upon drying, cellulose retains residual bound water within or between crystalline sheets, whereas pectin aggregates lose most of their internal water, resulting in matrix collapse.^[^ ^26^ ^]^ This difference stems from the nature of water–polymer interactions: cellulose forms strong hydrogen bonds that stabilize its structure and retain water, while pectin contains loosely bound water that is readily lost during desiccation, leading to structural breakdown. Building on these insights, we propose a mechanism underlying the desiccation‐induced polarity inversion of *S*_i_ (Figure 3c). Under hydrated conditions, cellulose microfibrils maintain a rigid, organized framework that facilitates cation thermodiffusion through hydroxyl‐mediated interactions. In contrast, the hydrated pectin matrix is more flexible and dispersed, promoting anion thermodiffusion via carboxylate groups. This structural contrast likely renders pectin a more efficient ion‐conducting channel than cellulose, leading to net anionic thermodiffusion during the first 0–12 h of drying (Figure 3c). As desiccated progresses (16 – 24 h), the pectin matrix collapses due to aggregation and dehydration^[^ ^26^ ^]^ thereby immobilizing ions and suppressing anion transport. Meanwhile, cellulose microfibrils retain structural integrity and residual water,^[^ ^26^ ^]^ allowing cation thermodiffusion to persist (Figure 3c). Consequently, cation thermodiffusion becomes dominant as drying continues.

To further examine the polarity inversion, we compared ion concentrations in the cold regions of fresh and 24‐h desiccated leaves. Ion chromatography revealed that in fresh leaves, anion concentrations (NO_3_^−^, Cl^−^, and PO_4_^3−^) exceeded that of cation (K^+^). In contrast, this trend was reversed in the desiccated leaf: cation concentrations surpassed anion levels (Figure 3d; Figures S13–S16, Supporting Information). Furthermore, NO_3_^−^ and Cl^−^ were more concentrated on the hot side than the cold side after desiccation, whereas such asymmetry was not observed in fresh leaves (Table S1; Figures S13–S16, Supporting Information). The dominant cation was K^+^, which is lighter and more mobile than the predominant anion PO_4_^3−^. This difference in mobility helps explain the bipolar thermovoltage behavior observed after 12 h of desiccation (Figure 3a).

Desiccation significantly alters the water content of the leaf, which in turn impacts ion diffusion.^[^ ^27^ ^]^ Water content (WC, %) was estimated by measuring the difference in leaf weight before and after desiccation using the following equation:

(2) $$Water\;content\left(\%\right)=\frac{original\;weight-desiccated\;weight}{desiccated\;weight}\times 100$$

A fresh 2 cm × 1 cm leaf contained 88.2 ± 0.2 % water, which decreased nearly tenfold to 8.5 % after 24 h of desiccation (inset in Figure 3e). This loss of water correlated with reduced ion conductivity (σ, mS cm^−1^), as measured by impedance spectroscopy (see Methods for details). Conductivity decreased from 20 mS cm^−1^ in the fresh leaf to 0.59 mS cm^−1^ after 24 h of desiccation (Figure 3e).

### 2.6 Power Factor and Figure of Merit

The power factor (*PF*, µWm^−1^K^−2^), defined as *PF* = σ × *S_i_* ^2^, initially decreased sharply within the first 12 h of desiccation, followed by a dramatic increases beyond 12 h (Figure 3f). Notably, after 24 h, the *PF* value was two orders of magnitude higher than that of the fresh leaf. We also studied the impact of desiccation on the dimensionless figure of merit (*ZT*), defined as *ZT* = *PF*/*K*, where *K* (Wm^−1^K^−1^) is the thermal conductivity of the leaf. Detailed experimental procedures for *K* measurements are provided in Supporting Information. The measured *K* values for fresh and 24‐h desiccated leaves were 0.39 ± 0.003 and 0.30 ± 0.001 Wm^−1^K^−1^, respectively (**Table** **1**; Figure S24, Supporting Information), comparable to values reported for electrolyte‐infiltrated cellulosic membranes^[^ ^8^ ^]^ and ionic gels.^[^ ^19^, ^28^, ^29^ ^]^ Following 24 h of desiccation, the *ZT* increased by two orders of magnitude. The remarkable enhancement in both *PF* and *ZT* was primarily driven by the significant increase in the Seebeck coefficient (Table 1).

**Table 1:** Summary of thermal power (*S*), ion conductivity (σ), power factor (*PF*), thermal conductivity (*K*), and figure of merit (*ZT*) of leaves depending on the desiccation conditions.

Condition	*S* [mVK^−1^]	σ [mS cm^−1^]	*PF* [µWm^−1^K^−2^]	*K* [Wm^−1^K^−1^]	*ZT* at RT
Fresh	−0.62	20	0.78	0.39	4.86 × 10^−4^
24 h desiccation	28.65	0.59	59.30	0.30	× 10^−2^

### 2.7 Dielectric Capacitive Model

**Figure 4:**
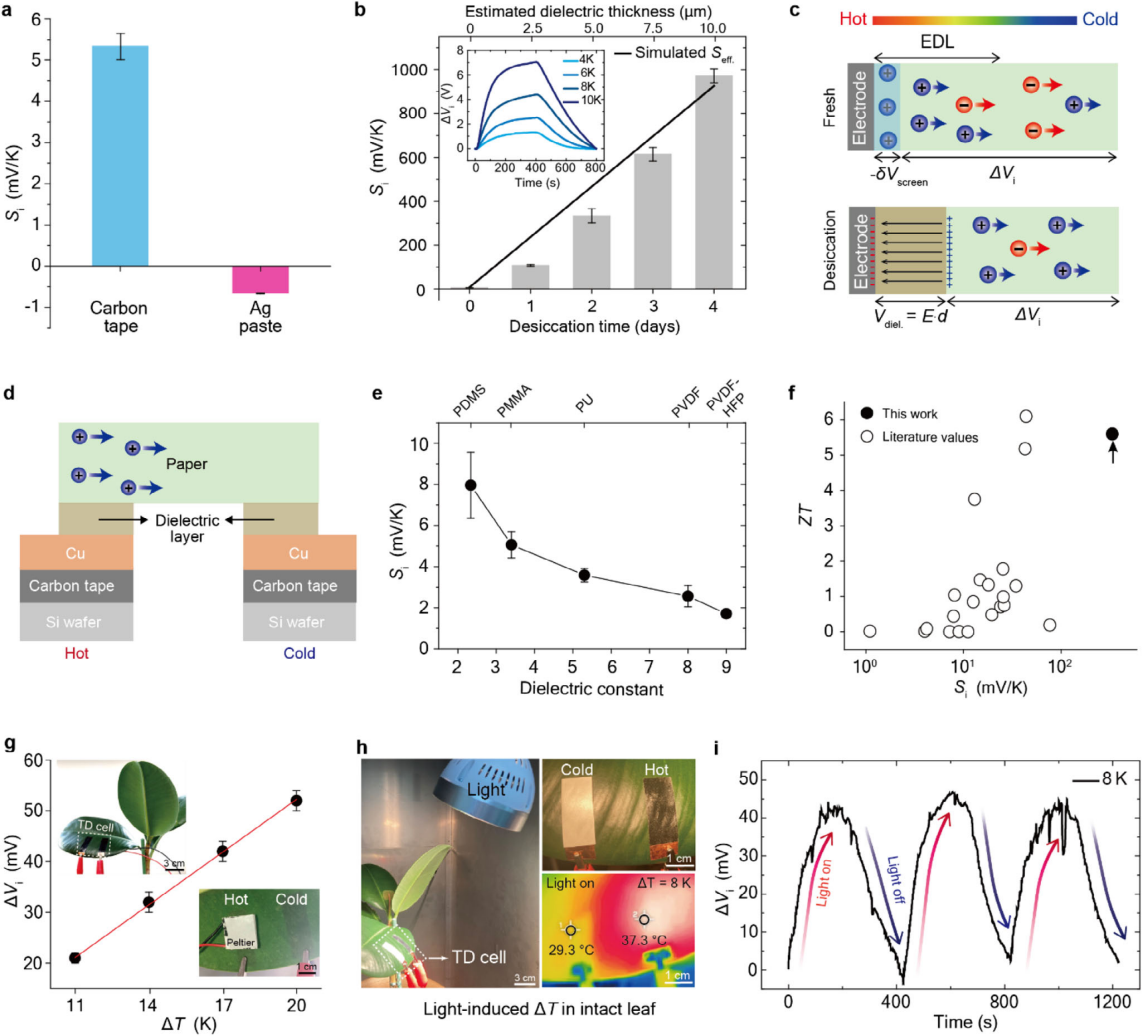
Strategies for enhancing thermopower and demonstration of non‐destructive, in vivo thermopower generation in a leaf. a) Comparison of ionic Seebeck coefficient (*S*_i_, mV K^−1^) in fresh leaves using carbon tape and Ag paste electrodes. b) *S*_i_ values for leaves with carbon tape electrodes as a function of desiccation time (1–4 days). Black line plot represents the simulated effective Seebeck coefficient (*S*_eff._). Inset: representative thermovoltage (Δ*V*_i_) curves for a 4‐day desiccated leaf, exhibiting an ultra‐high Δ*V*_i_ of ≈7 V at Δ*T* = 10 K. c) Schematic illustration showing the effect of a dielectric layer on Δ*V*_i_, comparing direct ion–electrode contact in fresh leaves (upper) with ion transport across an interfacial dielectric layer formed upon desiccation (lower). d) Device structure of the model thermodiffusion cell, consisting of cellulose soaked in 1 m NaOH, combined with polymeric dielectric layers (≈10 µm thickness). e) Correlation between *S*_i_ and the dielectric constant (ε) of the polymeric layers. The ε values for the polymers are: 9.0 (PVDF‐HFP; poly(vinylidene fluoride‐co‐hexafluoropropylene), 8.0 (PVDF; polyvinylidene fluoride), 5.3 (PU; polyurethane), 3.4 (PMMA; poly(methyl methacrylate)), and 2.3 (PDMS; polydimethylsiloxane). f) Comparison of *S*_i_ and figure‐of‐merit (*ZT*) values (at 298 K) for a 2‐day desiccated leaf (carbon tape electrodes) with those of various synthetic materials reported in the literature.^[^ ^7^, ^8^, ^28^, ^29^, ^34^, ^35^, ^36^, ^37^, ^38^, ^39^, ^40^, ^41^, ^42^, ^43^, ^44^, ^45^, ^46^, ^47^, ^48^, ^49^, ^50^, ^51^, ^52^ ^]^ g) Plot of *∆V*_i_ versus *∆T* measured in a live, intact leaf under Peltier‐induced heating. Inset: Photographs of the in vivo thermodiffusion (TD) cell setup. h) Left: Photograph of light‐induced *∆T* generation in intact leaf TD cell using light source (24 Wh). Upper right: Photograph showing assembled carbon tape electrodes, with one electrode covered in white paper to maintain a lower temperature. Lower right: Infrared thermographic image confirming *∆T* generation upon light exposure. i) *∆V*_i_ response recorded over three cycles of light on (heating) and off (cooling) in the intact leaf‐based TD cell. Error bars in (b, e, g) indicate the standard deviation obtained from five independent measurements for each data point.

To assess the influence of electrode material on the thermopower in leaves, we compared measurements using Ag paste and carbon tape electrodes. With Ag paste electrodes, fresh leaves exhibited n‐type thermopower, whereas carbon tape electrodes produced p‐type response (**Figure** **4**a). This polarity difference suggests that the cation affinity of the carbon tape enhances the detection of cationic thermodiffusion.^[^ ^30^, ^31^ ^]^ The effect of electrode materials on *S*_i_ polarity has been previously reported in other thermodiffucion systems.^[^ ^31^, ^32^ ^]^ We hypothesize that it may arise from differences in the structure of the electrical double layer (EDL) at the electrode‐electrolyte interface. The EDL, comprising a compact Stern layer and a surrounding diffuse layer, forms at both types of electrodes.^[^ ^33^ ^]^ However, carbon tape appears to adsorb fewer ions in the Stern layer than Ag paste, leading to reduced electrostatic screening and, consequently, a greater effective *S*_i_ (Figure 4a; Figure S25, Supporting Information).

The *S*_i_ of leaves with carbon tape electrodes increased steadily with desiccation time—without polarity inversion—reaching +971.5 mV K^−1^ after four days of drying (Figure 4b; Figure S26, Supporting Information). Under a moderate temperature gradient of 10 K, the resulting Δ*V*_i._ reached ≈7 V (inset in Figure 4b). This desiccation‐induced enhancement can be explained by changes in the EDL structure. In fresh leaves, a conventional Stern layer would form at the electrode‐electrolyte interface (Figure 4c, upper panel),^[^ ^33^ ^]^ introducing a screening effect (–δ*V*_screen_) that reduces the effective Δ*V*_i_ value. As desiccation proceeds, a dielectric layer—composed of dried cellulose—forms between the electrode and the internal electrolyte, effectively inhibiting direct Stern layer formation. Simultaneously, thermodiffusion‐driven surface charge redistribution induces capacitive polarization across this dielectric barrier, generating an additional interfacial potential (Figure 4c, lower panel). This polarization field (*E*, black arrows) contributes constructively to total thermovoltage and can be expressed as:

(3) $$V_{diel.}=\frac{\sigma \cdot d}{\varepsilon_{r}\cdot \varepsilon_{0}}$$

where *σ* is the effective surface charge density, *d* is the thickness of the dielectric layer, *ε*_r_​ is the relative permittivity of the dielectric, and *ε*_0_ is the vacuum permittivity. As desiccation progresses, this capacitive contribution becomes increasingly significant, amplifying the overall voltage output.

To better understand the capacitive contribution to thermopower, we developed a hybrid simulation model that combines the classical Poisson–Nernst–Planck (PNP) framework^[^ ^53^ ^]^ for ion thermodiffusion with an interfacial dielectric model that captures capacitive voltage generated at the electrode–electrolyte interface (see Supporting Information for details). Optical microscopic analysis revealed that the maximum thickness of the dried region reached ≈10 µm after 4‐day desiccation time (Figure S27, Supporting Information). Accordingly, the dielectric thickness *d*, representing the dried cellulose layer, was assumed to increase with desiccation time, reaching 10 µm after four days. The effective thermovoltage (*V*_eff._) is modeled as the sum of the interfacial dielectric potential (*V*_diel._) and the thermodiffusion potential from the bulk electrolyte (*V*_electrolyte_):

(4) $$V_{eff.}=V_{diel.}+V_{electrolyte}$$

Accordingly, the effective Seebeck coefficient (*S*_eff._) is defined as:

(5) $$S_{eff.}=\frac{V_{eff.}}{\Delta T}$$

The *V*_electrolyte_ obtained from the PNP model was 80 mV, in close agreement with the experimental value (Figure S26, Supporting Information). The *σ* value was determined self‐consistently during this calculation. The *ε*_r_​ was set to 3.0, representing the average of reported values for dried cellulose (2.5 to 3.5).^[^ ^54^ ^]^ Using *σ* = 2.44 × 10^−5^ C m^−2^ and *ε*_r_ = 3.0, the model yielded a best‐fit to experimental *S*_i_ value approaching *≈*970 mV K^−1^ at a dielectric thickness of 10 µm (Figure 4b). To access the role of dielectric properties, *ε*_r_ was varied from 1 to 5. Notably, values in the range of 2.5 to 3.5 yielded simulated *S*_eff_ values that best aligned with experiment (Figure S28, Supporting Information), consistent with reported *ε*_r_​ values for dried cellulose (2.5–3.5).^[^ ^54^ ^]^ These results support the validity of this model in capturing the desiccation‐dependent enhancement in *S*_i_.

To experimentally validate the model, we constructed thermodiffusion cells using cellulose paper soaked in 1 m NaOH solution, with and without a polymeric dielectric interlayer (Figure 4d). This setup enabled direct evaluation of the dielectric layer's effect on cation thermodiffusion. In the absence of a dielectric layer, Δ*V*_i_ increased with temperature but showed considerable fluctuations during isothermal periods (Figure S29a, Supporting Information). In contrast, introducing a ≈10 µm‐thick polydimethylsiloxane (PDMS) layer between the cellulose and the electrode enhanced both Δ*V*_i_ and stability of Δ*V*_i_ under constant temperature (Figure S29b, Supporting Information). These results support the hypothesis that the dielectric layer suppresses the electrostatic screening effect of the Stern layer, thereby stabilizing and amplifying the thermodiffusion signal. According to Equation (3), a lower *ε*_r_ leads to a larger interfacial dielectric *V*_diel._, enhancing the total thermovoltage. To further confirm this effect, we tested polymeric interlayers with varying *ε*_r_ while maintaining a fixed thickness of ≈10 µm. As shown in Figure 4e, the use of low‐*ε*_r_ PDMS (≈2.3) resulted in an *S*_i_ value ≈10 times greater than that of the control (bare paper). This finding indicates that lower *ε*_r_ leads to increased thermopower, consistent with the model prediction that capacitive contributions from the dielectric layer augments Δ*V*_i_. After 2 days of drying, the *ZT* value reached ≈5.6 (at 298 K), surpassing many synthetic materials (Figure 4f; Table S3). See Figure S30 and Table S4 for the *K* and σ values of desiccated leaf samples with carbon tape electrodes. This exceptionally high *ZT* is primarily attributed to the large *S*_i_, despite the relatively low ionic conductivity. These results highlight that electrode material plays a role in modulating both the polarity of dominant charge carriers and the desiccation effect on thermopower performance of leaf.

### 2.8 In Vivo Thermoelectric Assay in Leaf

To evaluate the feasibility of observing ionic thermoelectric behavior in vivo, we conducted a non‐destructive assay using a live, intact leaf. As illustrated in the inset of Figure 4g, carbon tape electrodes were gently attached to both sides of the living leaf surface, and a Peltier element was applied to only one electrode to create a temperature gradient. The resulting Δ*V*_i_ increased steadily with temperature, yielding a *S*_i_ of +3.46 ± 0.05 mV K^−1^ (Figure 4g). This value is comparable to +5.35 ± 0.32 mV K^−1^ measured in cut‐leaf specimen, confirming that the ionic Seebeck effect can be reliably achieved in living tissue.

To examine whether the in vivo thermopower generation can be achieved with light irradiation‐induced heat, we designed a light‐driven setup (Figure 4h). One electrode was left exposed with black carbon tape to absorb incident light and act as the hot side, while the opposing electrode was covered with white paper to reduce heat absorption and maintain a cooler temperature (Figure 4h, upper right). Upon illumination from a ≈30 cm distance using a 24 Wh light source (Figure 4h, left), an 8 K temperature gradient developed across the leaf, as confirmed by IR imaging (Figure 4h, lower right). Repeated light on/off cycling at 200‐s intervals produced consistent Δ*V*_i_ signals of ≈45 mV (Figure 4i). For the long‐term stability test, 30 consecutive photothermal cycles were performed, and the thermovoltage was consistently measured with an average value of 42.1 ± 1.5 mV (Figure S31, Supporting Information). In addition, chlorophyll activity was evaluated by comparing leaves with attached electrodes after prolonged measurements to fresh, untreated leaves. The results showed no significant degradation in chlorophyll activity, indicating that the leaf tissue remained physiologically active throughout the extended measurement period (Figure S32, Supporting Information). These findings demonstrate that living leaves can function as ionic thermoelectric systems, converting light‐induced temperature gradients into measurable voltages—without the need for invasive processing or tissue disruption.

## 3 Conclusion

In this study, we demonstrated a high‐performance, environmentally friendly thermoelectric system based on natural leaves by harnessing the ionic Seebeck effect. Controlled desiccation and electrode selection led to substantial enhancements in thermopower and energy conversion efficiency. Notably, a desiccated leaf equipped with carbon tape electrodes achieved a figure of merit (*ZT*) of ≈5.6 at 298 K after two days, and an ionic Seebeck coefficient (*S*_i_) of 971 mV K^−1^ with a thermovoltage of ≈7 V under a modest 10 K temperature gradient after four day‐desiccation. This enhancement is explained by a dielectric capacitive model, in which the desiccated leaf surface functions as a natural dielectric layer that amplifies voltage output. This model was supported by simulations and validated experimentally using paper‐based thermodiffusion cells with synthetic dielectric layers. Furthermore, we achieved repeatable thermovoltage generation in intact, living leaves by employing a non‐destructive electrode integration strategy and leveraging light‐induced temperature gradients—demonstrating in vivo functionality without compromising tissue integrity. These findings reveal the untapped potential of natural, biodegradable materials as efficient and sustainable platforms for thermoelectric energy harvesting. The use of non‐toxic, eco‐compatible components makes this approach particularly attractive for future green energy technologies. Moving forward, investigating the role of leaf microstructure in ionic thermopower may offer new opportunities in biomimetic thermoelectrics and next‐generation energy systems.

## 4 Experimental Section

### Extraction of Cellulose and Pectin from Leaves

All reagents were used as supplied unless otherwise specified. NaOH, HCl, ethanol and NaClO_2_ were purchased from Daejung. Water was purified using an Aqua MAX‐Basic System (deionized water with a resistivity of ≈18.2 MΩ·cm). The extraction of cellulose from *Ficus elastica* leaves was conducted following a previously reported procedure (Figure S3a, Supporting Information).^[^ ^18^ ^]^ Leaves were dried for 3 days, then ground and treated with 4 % NaOH at 125 °C for 12 h. A bleaching treatment was carried out using 1.7 w/v% NaClO_2_ at pH 4.5 and 125 °C for 6 h. The resulting products were washed with 1 % HCl and deionized (DI) water. For the extraction of pectin (Figure S3b, Supporting Information),^[^ ^17^ ^]^ dried leaves were shredded into pieces and then crushed using a mixing grinder. The shredded leaves were treated with an alkaline solution (adjusted to pH 8 using NaOH) at 60 °C for 3 h. Supernatants were isolated via centrifugation at 5000 rpm for 10 min. To remove small molecules (ions, monosaccharides, free amino acids, or free phenols), the supernatants were precipitated by adding 10 mL of ethanol. After a second centrifugation at 5000 rpm for 10 min, the resulting precipitates were collected and resuspended in 10 mL of DI water.

### Cell Fabrication and Ionic Thermovoltage Measurements

A 1 cm × 2 cm rectangular section was cut from a leaf, and Ag paste electrodes were applied to both ends (Figure S5, Supporting Information). A temperature gradient (∆*T*) across the leaf was created by heating one end with a Peltier device, while the other end remained at ambient temperature. The Δ*T* applied by the Peltier varied from 2 to 10 K, with a heating duration of 200 s at each Δ*T*. The corresponding ionic thermovoltage (Δ*V*_i_) curves were recorded.

### Ionic Thermovoltage Data Analysis

Due to the presence of multiple ion species in the leaf, the initial measured voltage does not start at 0 mV, even when there was no temperature difference (Δ*T*) between the electrodes. However, once a constant temperature gradient was established, the voltage change (Δ*V*) follows a uniform trend, as illustrated in Figure S6a (Supporting Information). A baseline was drawn at the point where the Δ*T* = 0 K (Figure S6a, Supporting Information) and was used to determine the change in Δ*V* as a function of Δ*T*, as depicted in Figure S6b (Supporting Information).

### Optical Microscopy Analysis

Leaves were cut into 0.5 cm × 0.5 cm small pieces and embedded in 3% agarose for 3 h.^[^ ^15^, ^16^ ^]^ The resulting samples were then sectioned into 300 µm‐thick slices using a microtome (RMC). The prepared ultrathin sections were observed under an optical microscope. In addition, to visualize the progression of the dried region within the leaf during desiccation, leaf samples subjected to 1–4 days of drying were embedded in 3% agarose for 15 min, sectioned using a microtome, and subsequently imaged via microscopy to obtain cross‐sectional images. See Figure S27 (Supporting Information) for data.

### Stain Method and Intensity Analysis

To stain pectin and cellulose, leaf sections were immersed in 0.04% aqueous solutions of Ruthenium Red and Congo Red, respectively, for 30 min.^[^ ^15^, ^16^ ^]^ After thorough rinsing with deionized water, the samples were examined microscopically.

### Ion Chromatography

A leaf subjected to a Δ*T* of 10 K for 300 s was cut into hot and cold sections for ion chromatography analysis. ≈1.0 g of leaf sample was leached in 100 mL of distilled water by shaking at room temperature for 3 h. The solvent used for ion extraction was a 3.2 mM Na_2_CO_3_ and 1.0 mM NaHCO_3_ mixture in deionized (DI) water. The injection volume for the ion chromatography column was 20 µL. Anions and cations were measured using the Metrohm 930 Complex IC Flex and Metrohm 883 Basic IC plus instruments, respectively. For anion detection, the following species were analyzed: F^−^, Cl^−^, NO_2_^−^, Br^−^, NO^3−^, PO_4_^3−^, and SO_4_^2−^ (Figures S9, S11, Supporting Information). F^−^, NO_2_^−^, Br^−^, SO_4_^2−^, and I^−^ were below the detection limit. For cation detection, the following ions were analyzed: Li^+^, Na^+^, NH_4_^+^, K^+^, Ca^2+^, and Mg^2+^ (Figures S10, S12, Supporting Information). F⁻, NO₂⁻, Br⁻, SO₄^2^⁻, I⁻, and Li⁺ were not detected, and there were no significant concentration differences in Na^+^ and NH_4_^+^ between the hot and cold sections. The same procedure was applied to analyze desiccated leaf samples (Figures S13–S16, Supporting Information).

### Ion Conductivity

The ionic conductivity of the leaf was determined by measuring impedance using a potentiostat (MULTI AUTOLAB M204, Metrohm Autolab B.V.). Resistance (*R*, Ω) was obtained from impedance measurements, and ionic conductivity (*σ*, mS cm^−1^) was calculated as:^[^ ^8^ ^]^

(6) $$\sigma_{ion}=\frac{1}{R}\frac{l}{A}$$

where *σ*_ion_ was the ionic conductivity, *l* was the sample length, and *A* was the contact area.

### Thermal Conductivity

The thermal conductivity (*K*) was calculated using the following equation:

(7) $$K=\alpha \rho c_{p}$$

where *α* was the thermal diffusivity, *ρ* was the density, and *c*_p_ was the heat capacity.^[^ ^8^ ^]^ A Netzsch differential scanning calorimeter (DSC 204 F1 Phoenix) was used to measure heat capacity. The heat capacity of fresh and 24‐h desiccated leaves was determined to be 3.8 (±1.0%) and 3.4 (± 1.0%) J⋅g^−1^⋅K^−1^, respectively. A Netzsch laser flash apparatus (LFA 467) was used to measure thermal diffusivity. The thermal diffusivity of fresh and 24‐h desiccated leaves was measured as 0.14 (± 1%) and 0.12 (± 1%) mm^2^⋅s^−1^, respectively. Eight separate measurements were conducted to obtain the average value for each sample. Finally, the thermal conductivity of fresh and 24‐h desiccated leaves was calculated as 0.39 ± 0.003 and 0.30 ± 0.001 W⋅m^−1^⋅K^−1^, respectively.

### Simulation

The ionic thermodiffusion process in bulk electrolyte and dielectric capacitive voltage formation were described by coupling the Poisson–Nernst–Planck (PNP) equations with an electrostatic model for capacitive‐induced voltage. Nernst‐Plank equation for ionic flux *J*_i_ was described as:^[^ ^53^ ^]^

(8) $$J_{i}=-D_{i}\left(\nabla c_{i}+\frac{z_{i}e}{k_{B}T}c_{i}\nabla \phi +\frac{Q_{i}}{k_{B}T^{2}}c_{i}\nabla T\right)$$

where *D_i_* was diffusion coefficient, *c_i_* was ion concentration, *z_i_* was valence, ϕ was electrical potential, and *Q_i_* was heat of transport. Poisson equation was calculated as:

(9) $$\nabla^{2}\phi =-\frac{e}{\varepsilon \varepsilon_{0}}\sum_{i}z_{i}c_{i}$$

where ε was cellulose dielectric constant in our work, and ε_0_ was vacuum permittivity. The PNP simulation assumed that a thermal gradient of 10 K was applied across a 10 mm domain filled with a dilute K⁺/PO₄^3^⁻ electrolyte. The K⁺ ion was assigned a non‐zero heat of transport, while PO₄^3^⁻ was assumed to remain thermally stationary. The obtained bulk electrolyte contribution (*V*_electrolyte_) by the PNP model was 80 mV. Separately, the dielectric‐induced interface potential was modeled as:

(10) $$V_{diel.}=\frac{\sigma \cdot d}{\varepsilon_{r}\cdot \varepsilon_{0}}$$

where *σ* was the effective surface charge density and *d* was the thickness of the dielectric layer. The *σ* value was determined self‐consistently during the PNP calculation. Thus, the effective Seebeck coefficient (*S*_eff._) was followed as:

(11) $$S=\frac{V_{diel.}+V_{electrolyte}}{\Delta T}$$

Based on film‐thickness estimation, the layer was modeled to increase from 0 to 10 µm over 0 to 4 days of ambient desiccation time. The parameter values used in simulation were shown in Table S2 (Supporting Information). To isolate the capacitive contribution to thermoelectric voltage, a model was simulated where only the hot‐side electrode accumulates ionic charge (σ = 2.44 × 10^−5^ C m^−2^) across a cellulose‐based dielectric layer. The induced interfacial voltage (*V* = *σ·d* / *ε*_r_ *·ε*_0_)​ was combined with a calculated electrolyte thermovoltage of 80 mV. The Seebeck coefficient (*S*_eff._ = *V*_total_/Δ*T*) was then calculated across various dielectric thicknesses and permittivity values (1.0 ≈ 5.0). Simulation results showed a strong inverse dependence of *S*_eff._ on ε_*r*_ (Figure S28, Supporting Information)​, with ε_*r*_ ≈ 3 yielding the best fit to experimental values approaching 971 mV K^−1^ at 10 µm.

### Fluorescence Imaging Analysis

The upper epidermis of control fresh leaves and leaves subjected to photothermal treatment were imaged using a CQ1 (YOKOGAWA) confocal microscope. Leaf discs (1 × 1 cm) were excised and incubated in 0.5 m Mannitol (pH 5.8) containing 0.05% (w/v) Calcofluor White for 5 min. Leaf discs were then rinsed twice in 0.5 m Mannitol. Fluorescence images were acquired using excitation wavelengths of 405 nm (calcofluor; emission 447 nm) and 640 nm (chlorophyll autofluorescence; emission 685 nm). Chloroplasts were counted automatically using the CQ1 software. Chloroplast density was calculated as chloroplasts per mm^2^ from three independent fields.

## Conflict of Interest

The authors declare no conflict of interest.

## Supporting information



Supporting Information

## Data Availability

The data that support the findings of this study are available in the supplementary material of this article.
