# Supplementary material for: High Ionic Seebeck Effect in Natural Leaves
Source: Adv Mater. 2025 Jul 26;37(41):e10413. doi: 10.1002/adma.202510413 (PMC12531751; doi:10.1002/adma.202510413)
Supplement: Supplementary file 1 — Supporting Information [file ADMA-37-e10413-s001.pdf]

ADVANCED MATERIALS

Supporting Information

for *Adv. Mater.*, DOI 10.1002/adma.202510413

High Ionic Seebeck Effect in Natural Leaves

*Hungu Kang, Hongwoo Lee, Cheljong Hong, Jiung Jang, Sahar Ayachi, Xin He, Pil Joon Seo, Alois Würger and Hyo Jae Yoon**

Supporting Information

High Ionic Seebeck Effect in Natural Leaves

*Hungu Kang, Hongwoo Lee, Cheljong Hong, Jiung Jang, Sahar Ayachi, Xin He, Pil Joon Seo, Alois Würger, and Hyo Jae Yoon**

This supporting information includes:

1. Supporting Figures (Figures S1 to S32)
2. Supporting Tables (Tables S1 to S4)

Supporting Figures

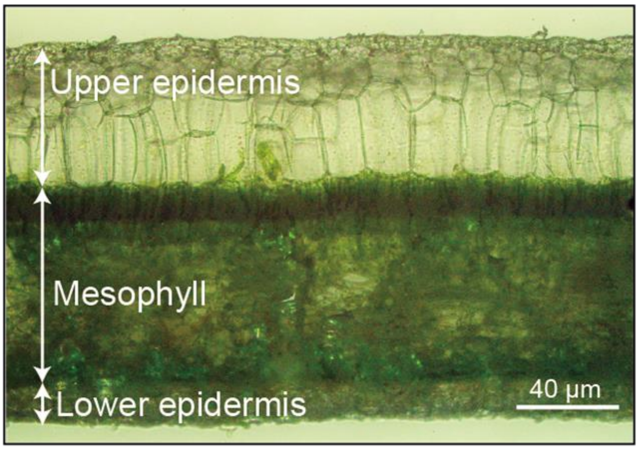

Figure S1. Microscopic image of the cross-sectional structure of the leaf, showing the upper and lower epidermis and mesophyll layers.

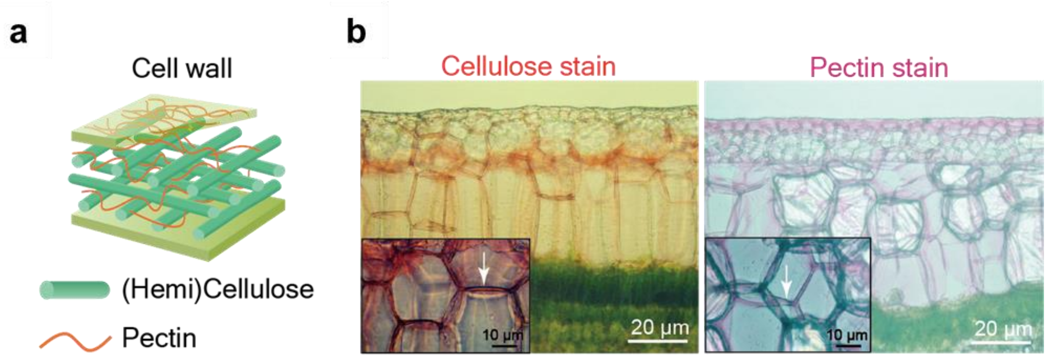

Figure S2. a) Schematic illustrating the plant cell wall composition, primarily consisting of (hemi)cellulose and pectin. b) Microscopic images of stained leaf cross-sections, with Congo Red (cellulose) and Ruthenium Red (pectin). The presence of each polymer is confirmed by red and purple coloration, respectively, as indicated by white arrows in the magnified insets.

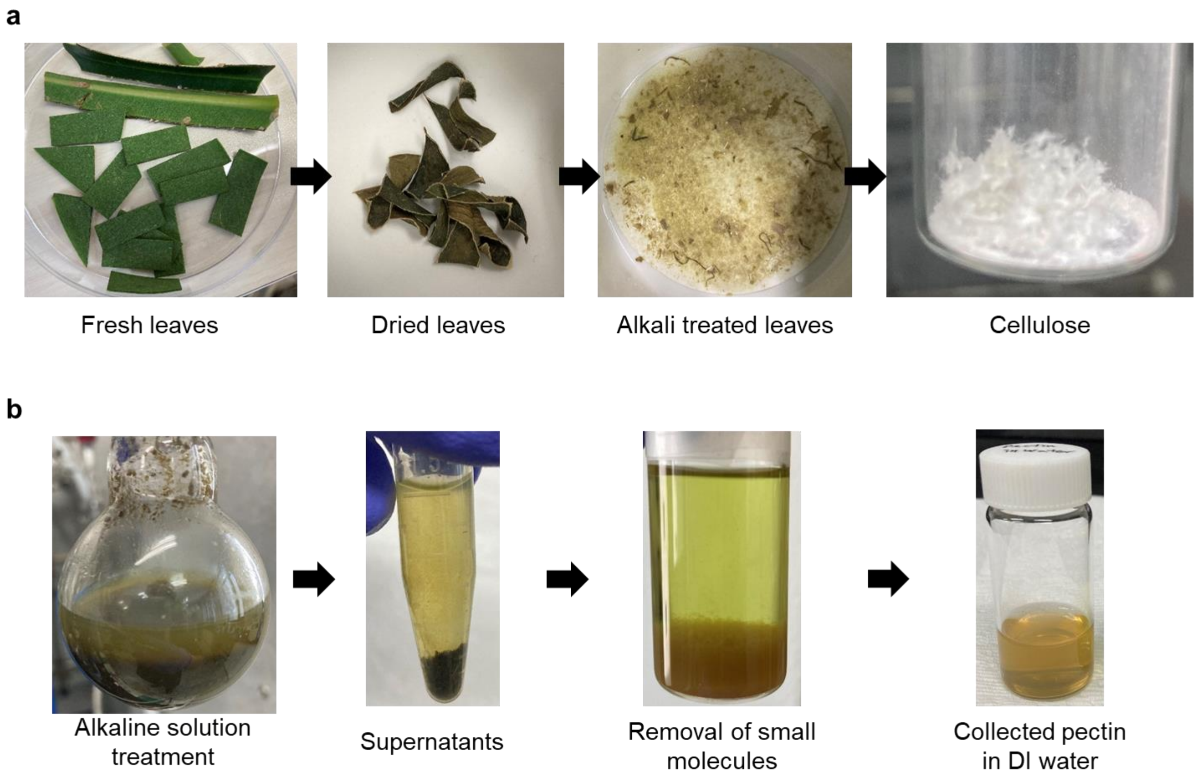

Figure S3. Extraction procedures for a) cellulose and b) pectin from leaves.

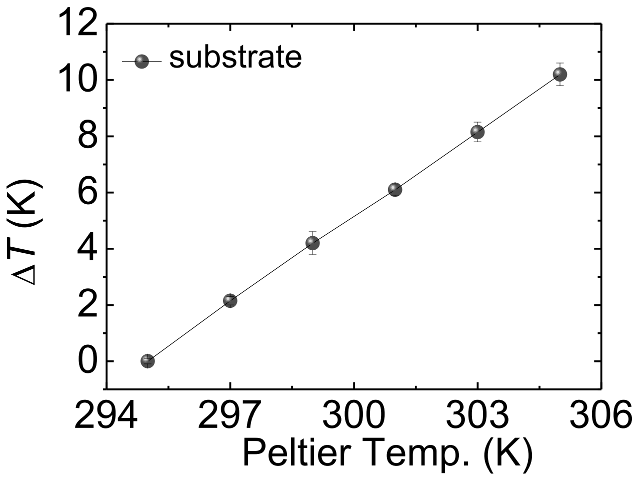

Figure S4. Plot of temperature differential (ΔT, K) of substrate (doped Si wafer) as a function of Peltier temperature.

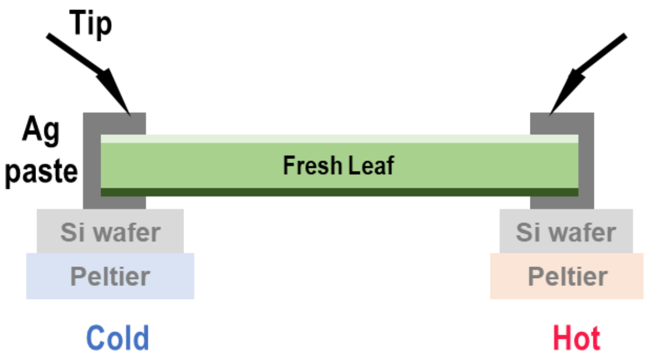

Figure S5. Schematic of the electrode-leaf-electrode thermodiffusion cell used to measure ionic thermovoltage (ΔV_i) under an applied temperature differential (ΔT).

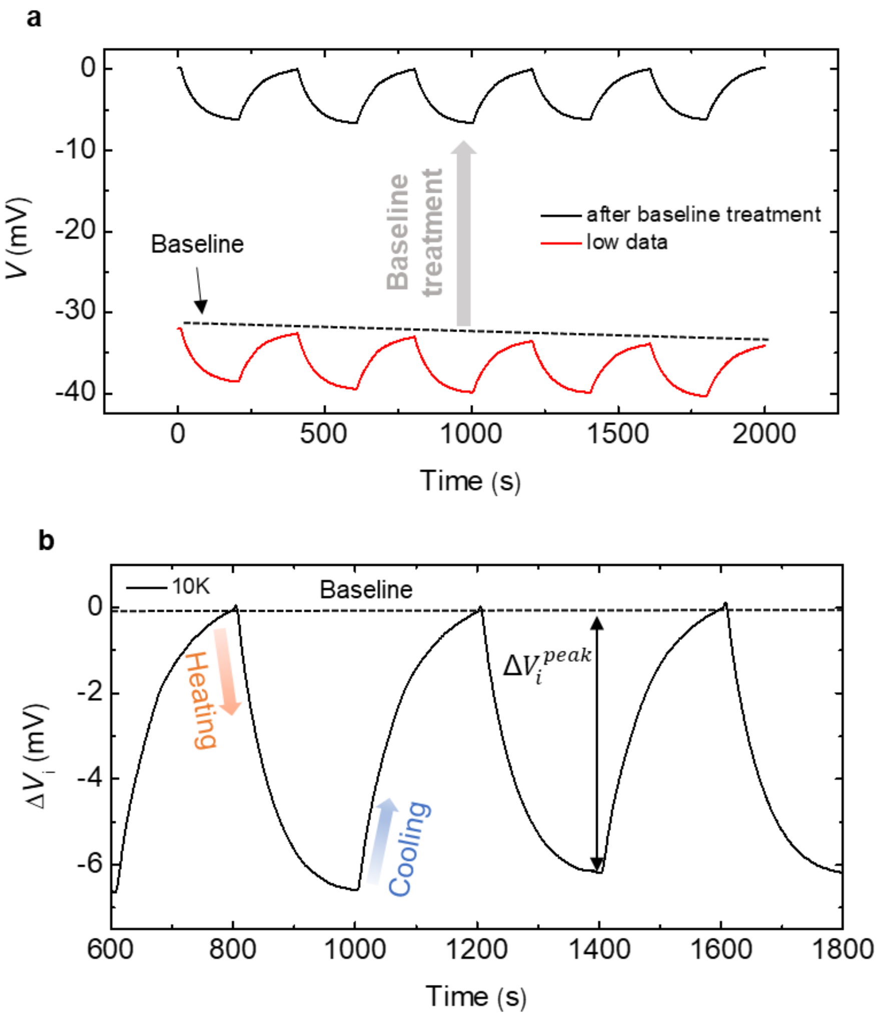

Figure S6. a) Raw open-circuit voltage data and baseline-corrected ionic thermovoltage (ΔV_i, mV). b) Representative ΔV_i curve in response to ΔT after baseline correction.

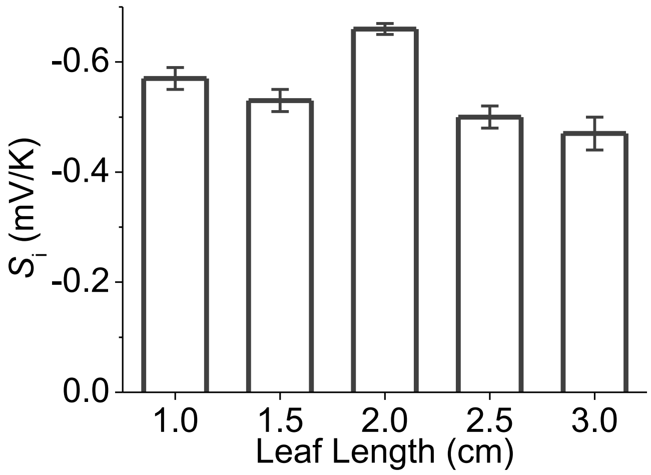

Figure S7. Plot of ionic Seebeck coefficient (S_i, mV/K) against the different leaf length. The different leaf lengths had no significant influence on S_i.

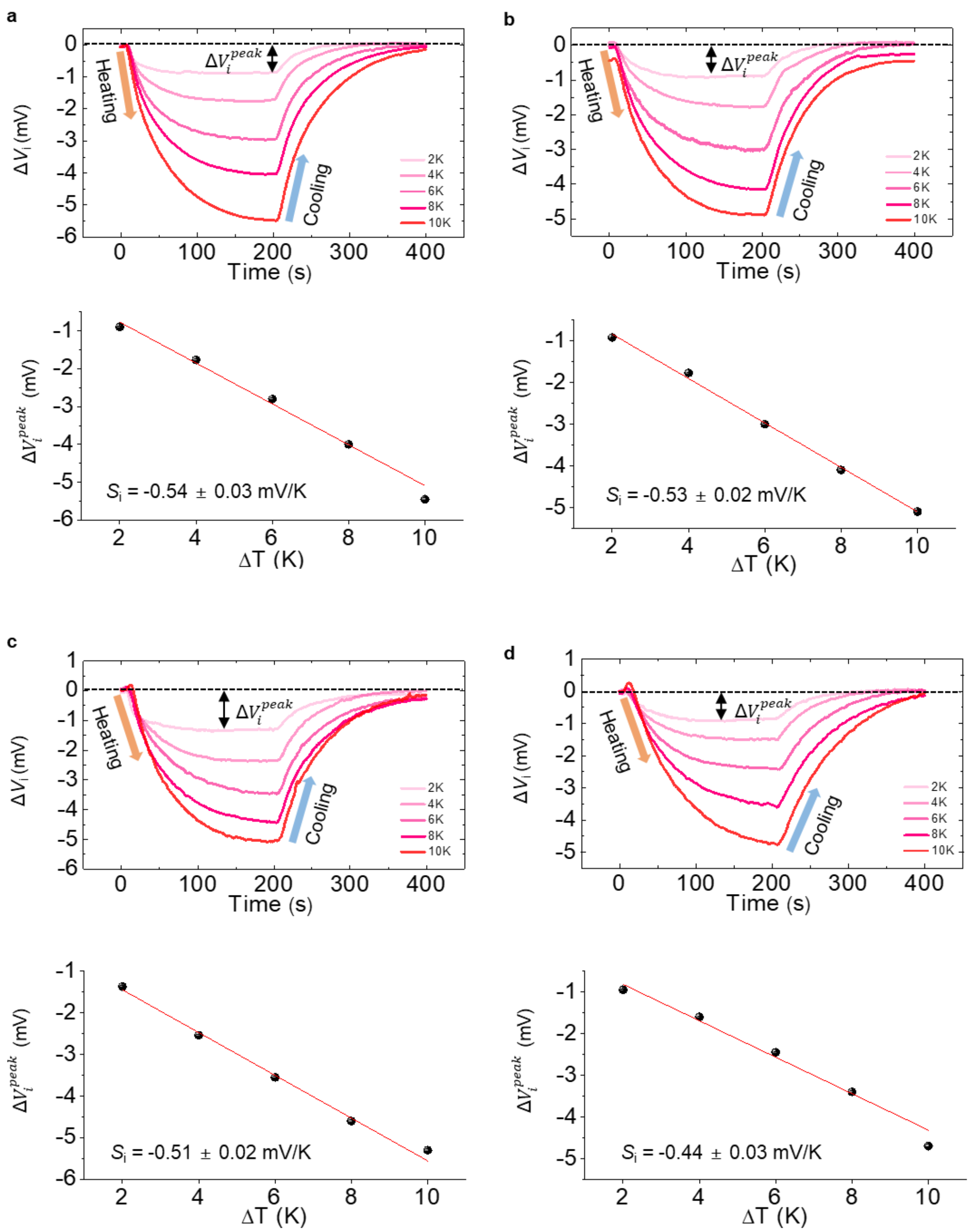

Figure S8. Effect of leaf length on ionic thermovoltage. Measured ΔV_i (mV) curves and corresponding $\Delta \boldsymbol{V_i^{peak}}$ plots as a function of time for fresh leaves with different dimensions: a) 1.0 cm × 1.0 cm, b) 1.0 cm × 1.5 cm, c) 1.0 cm × 2.5 cm, and d) 1.0 cm × 3.0 cm. $\Delta \boldsymbol{V_i^{peak}}$ represents the peak thermovoltage observed upon heating at each temperature difference (ΔT).

a

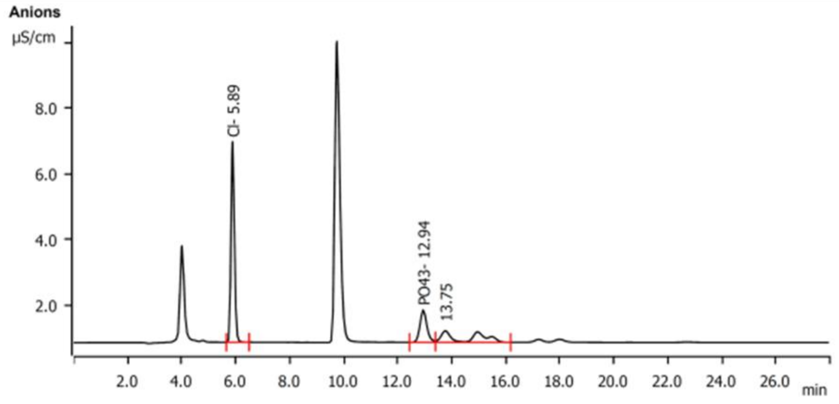

Peak number	Retention time	Area	Height	Concentration	Component name
	min	(µS/cm) x min	µS/cm	ppm	
1	5.885	0.8741	6.089	3.315	Cl-
2	12.940	0.2938	0.982	4.110	PO43-

b

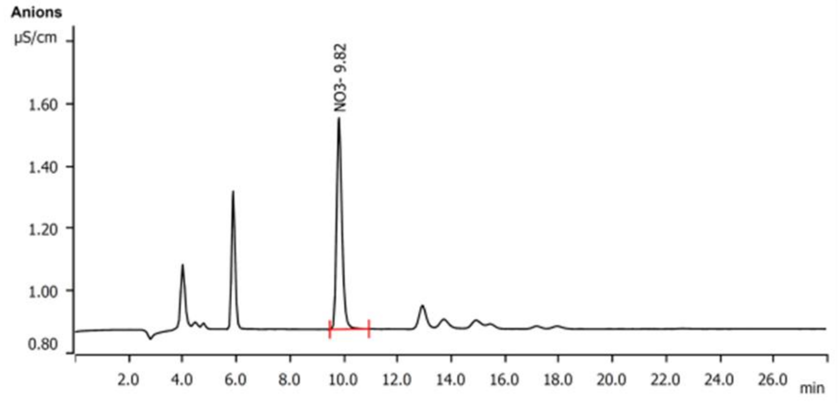

Peak number	Retention time	Area	Height	Concentration	Component name
	min	(µS/cm) x min	µS/cm	ppm	
1	9.817	0.1550	0.678	1.213	NO3-

Figure S9. Ion chromatogram data displaying anion separation in the cold-side sample of a fresh leaf: a) detection of Cl^- and PO_4^{3-}; b) detection of NO_3^-.

a

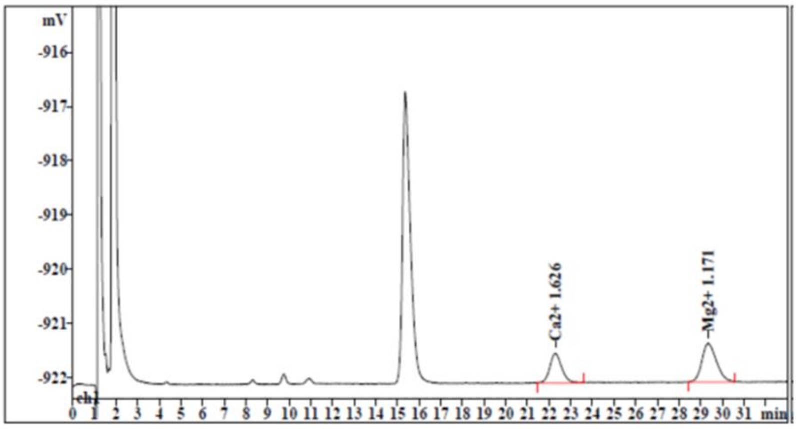

Quantitation method: Custom

No	Retention min	Height mV	Area mV*sec	Conc. mg/L	Name
1	22.29	0.54	20.963	1.626	Ca2+
2	29.36	0.71	33.425	1.171	Mg2+

b

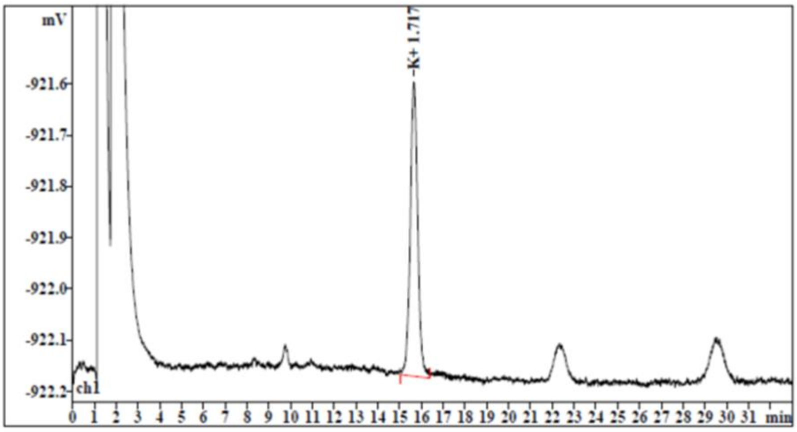

Quantitation method: Custom

No	Retention min	Height mV	Area mV*sec	Conc. mg/L	Name
1	15.66	0.57	13.615	1.717	K+

Figure S10. Ion chromatogram data displaying cation separation in the cold-side sample of a fresh leaf: a) detection of Ca^{2+} and Mg^{2+}; b) detection of K^{+}.

a

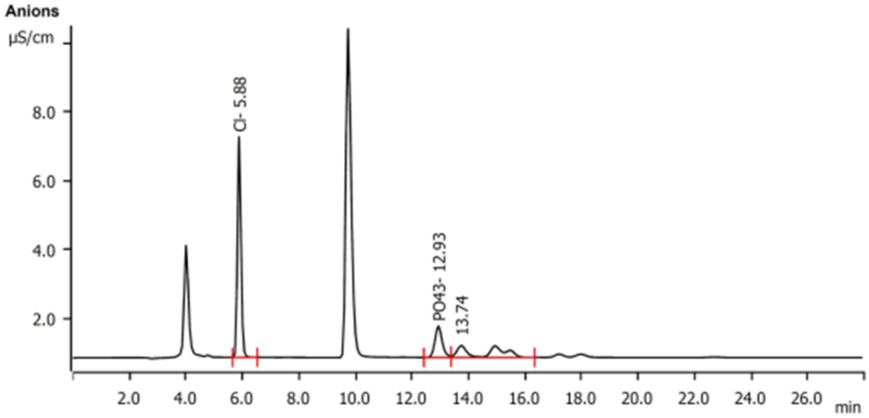

Peak number	Retention time	Area	Height	Concentration	Component name
	min	(µS/cm) x min	µS/cm	ppm	
1	5.880	0.9188	6.392	3.476	Cl-
2	12.930	0.2730	0.909	3.842	PO43-

b

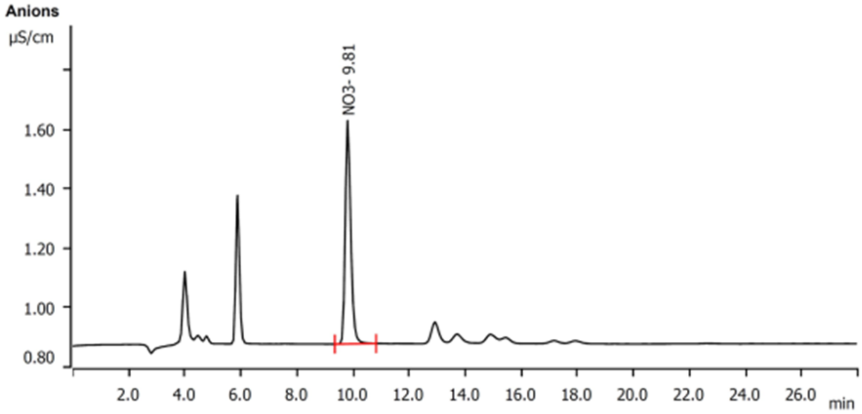

Peak number	Retention time	Area	Height	Concentration	Component name
	min	(µS/cm) x min	µS/cm	ppm	
1	9.807	0.1712	0.752	1.331	NO3-

Figure S11. Ion chromatogram data displaying anion separation in the hot-side sample of a fresh leaf: a) detection of Cl^- and PO_4^{3-}; b) detection of NO_3^-.

a

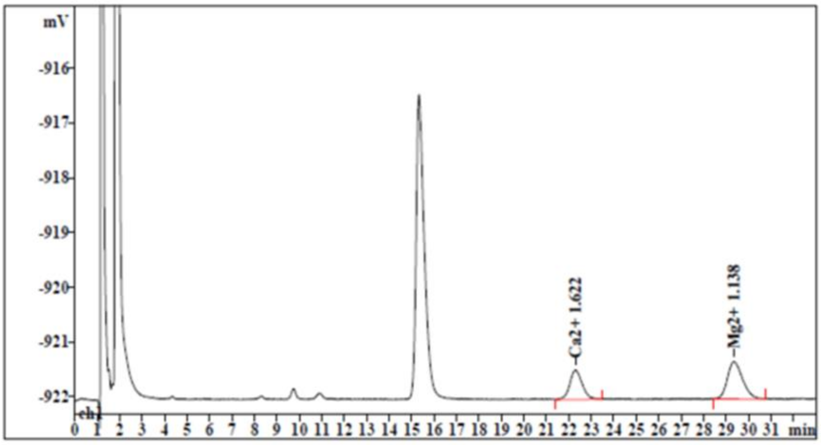

Quantitation method: Custom

No	Retention min	Height mV	Area mV*sec	Conc. mg/L	Name
1	22.30	0.54	20.906	1.622	Ca2+
2	29.36	0.68	32.502	1.138	Mg2+

b

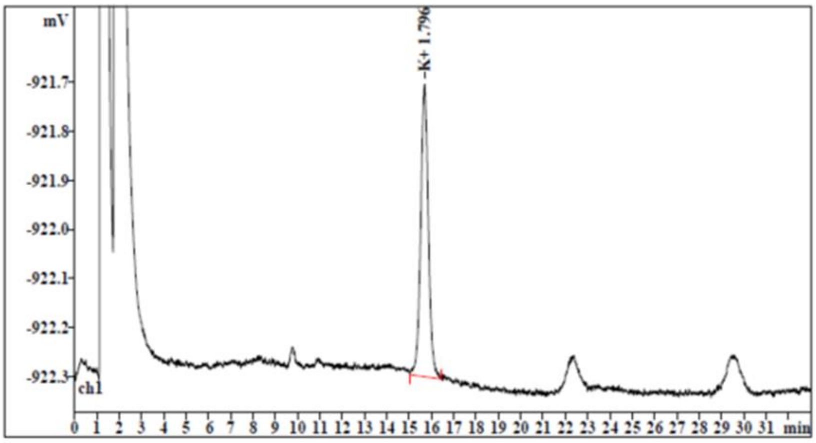

Quantitation method: Custom

No	Retention min	Height mV	Area mV*sec	Conc. mg/L	Name
1	15.69	0.59	14.237	1.796	K+

Figure S12. Ion chromatogram data displaying cation separation in the hot-side sample of a fresh leaf: a) detection of Ca^{2+} and Mg^{2+}; b) detection of K^{+}.

a

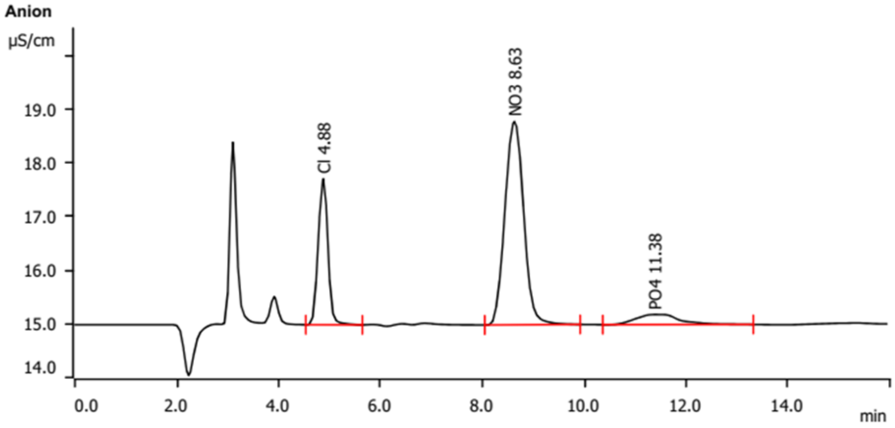

Peak number	Retention time	Area	Height	Concentration	Component name
	min	(µS/cm) x min	µS/cm	ppm	
1	4.878	0.6044	2.718	2.967	Cl
2	8.627	1.6188	3.784	14.895	NO3
3	11.375	0.1865	0.188	4.100	PO4

b

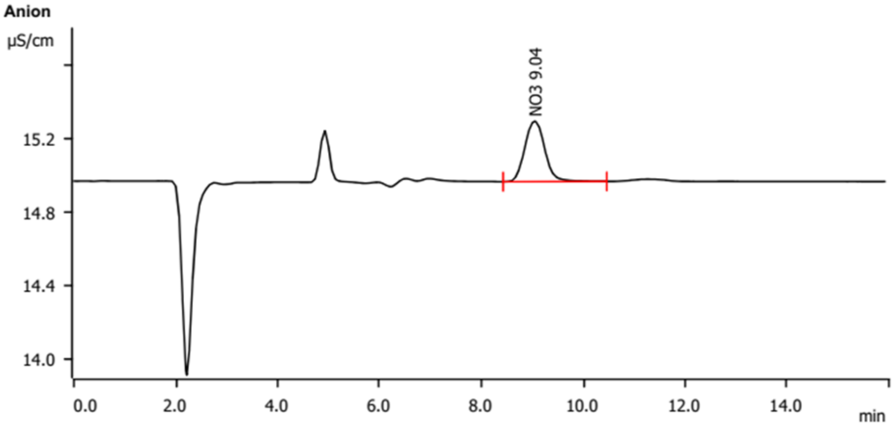

Peak number	Retention time	Area	Height	Concentration	Component name
	min	(µS/cm) x min	µS/cm	ppm	
1	9.043	0.1494	0.328	1.377	NO3

Figure S13. Ion chromatogram data displaying anion separation in the cold-side sample of a 24-hour desiccated leaf: a) detection of Cl^- and PO_4^{3-}; b) detection of NO_3^-.

a

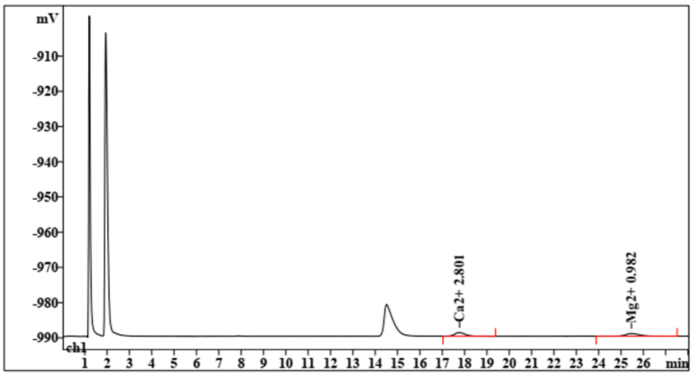

Quantitation method: Custom

No	Retention min	Height mV	Area mV*sec	Conc. mg/L	Name
1	17.77	1.03	35.270	2.801	Ca2+
2	25.46	0.74	33.530	0.982	Mg2+
3	28.00	1.77	68.800	3.783	

b

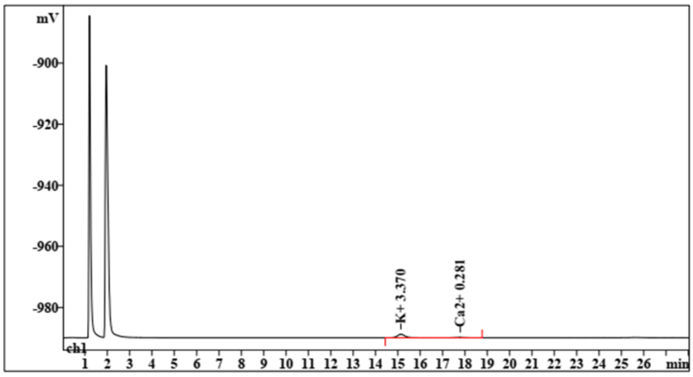

Quantitation method: Custom

No	Retention min	Height mV	Area mV*sec	Conc. mg/L	Name
1	15.13	1.16	28.689	3.370	K+
2	17.78	0.13	5.298	0.281	Ca2+
2	28.00	1.29	33.987	3.651	

Figure S14. Ion chromatogram data displaying cation separation in the cold-side sample of a 24-hour desiccated leaf: a) detection of Ca^{2+} and Mg^{2+}; b) detection of K^{+}.

a

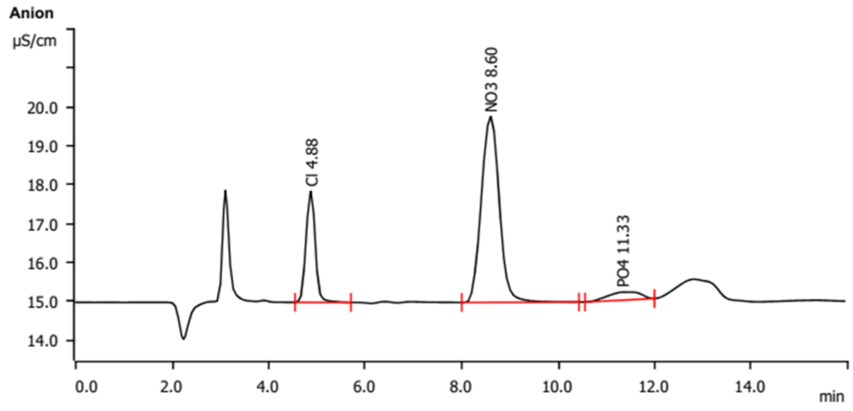

Peak number	Retention time	Area	Height	Concentration	Component name
	min	(µS/cm) x min	µS/cm	ppm	
1	4.875	0.6377	2.853	3.129	Cl
2	8.597	2.0619	4.777	18.971	NO3
3	11.328	0.1597	0.206	3.546	PO4

b

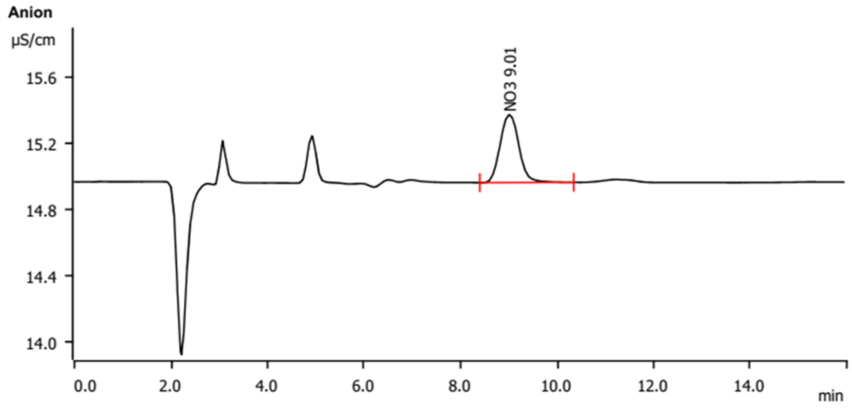

Peak number	Retention time	Area	Height	Concentration	Component name
	min	(µS/cm) x min	µS/cm	ppm	
1	9.008	0.1842	0.409	1.697	NO3

Figure S15. Ion chromatogram data displaying anion separation in the hot-side sample of a 24-hour desiccated leaf: a) detection of Cl^- and PO_4^{3-}; b) detection of NO_3^-.

a

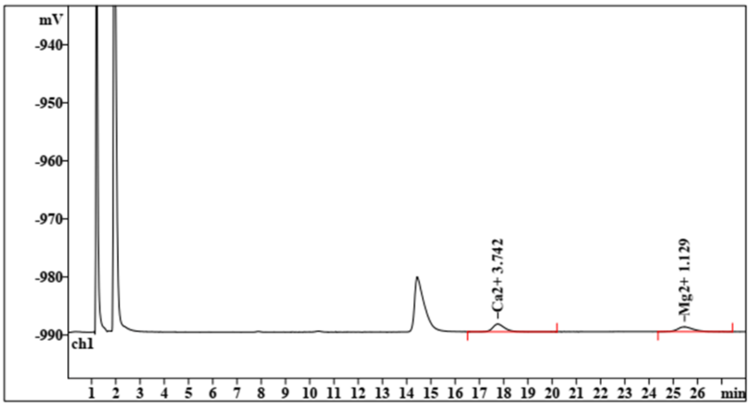

Quantitation method: Custom

No	Retention min	Height mV	Area mV*sec	Conc. mg/L	Name
1	17.76	1.33	46.405	3.742	Ca2+
2	25.46	0.82	37.982	1.129	Mg2+
2	28.00	2.14	84.387	4.872	

b

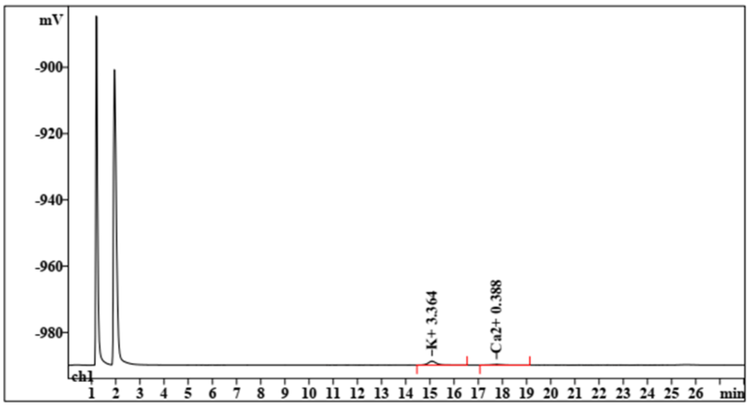

Quantitation method: Custom

No	Retention min	Height mV	Area mV*sec	Conc. mg/L	Name
1	15.08	1.17	28.636	3.364	K+
2	17.76	0.17	6.572	0.388	Ca2+
2	28.00	1.34	35.209	3.751	

Figure S16. Ion chromatogram data displaying cation separation in the hot-side sample of a 24-hour desiccated leaf: a) detection of Ca^{2+} and Mg^{2+}; b) detection of K^{+}.

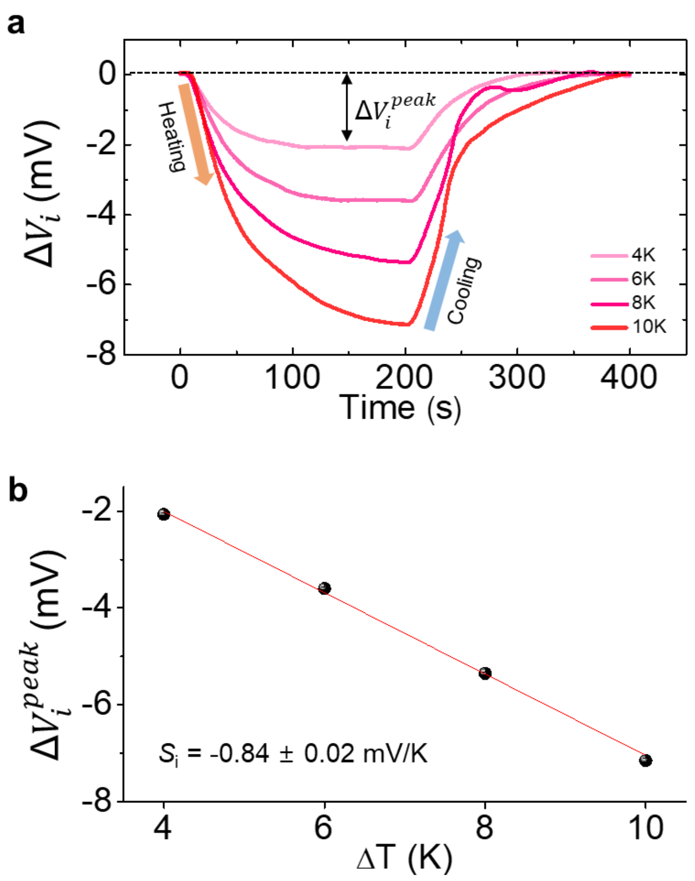

Figure S17. Effect of vein direction on ionic thermovoltage. a) Measured ionic thermovoltage (ΔV_i, mV) curves as a function of time for a fresh leaf with parallelly aligned veins. b) Plot of $\boldsymbol{\Delta V_i^{peak}}$ as a function of ΔT. $\boldsymbol{\Delta V_i^{peak}}$ represents the peak ΔV_i observed upon heating at each ΔT.

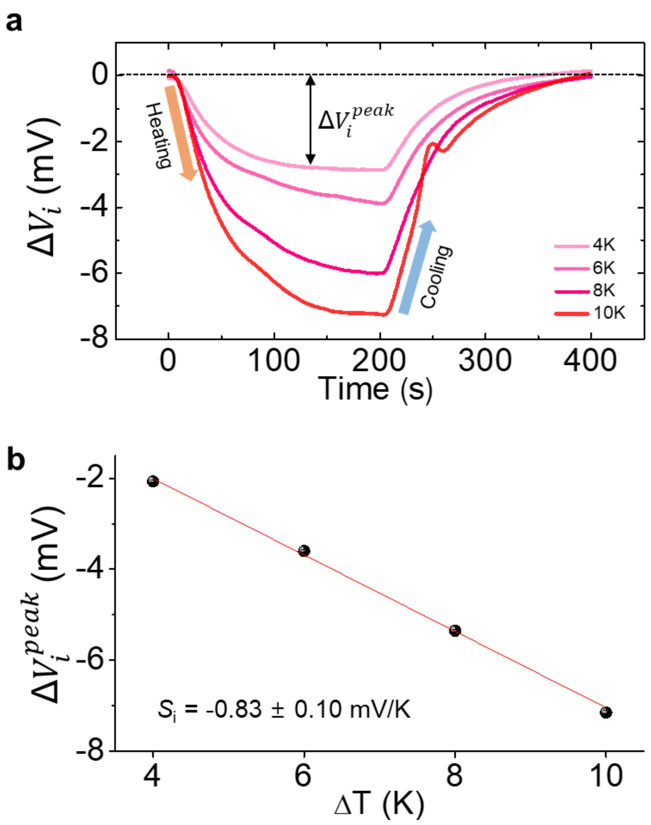

Figure S18. Effect of vein direction on ionic thermovoltage. a) Measured ionic thermovoltage (ΔV_i, mV) curves as a function of time for a fresh leaf with perpendicularly aligned veins. b) Plot of $\Delta \boldsymbol{V}_{\boldsymbol{i}}^{\boldsymbol{peak}}$ as a function of ΔT. $\Delta \boldsymbol{V}_{\boldsymbol{i}}^{\boldsymbol{peak}}$ represents the peak thermovoltage observed upon heating at each ΔT.

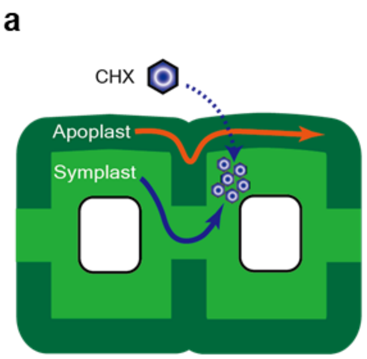

Figure S19. Schematics of a) symplast and b) apoplast inhibition process using cycloheximide (CHX) and $CuSO_4/K_4[Fe(CN)_6]$ as the symplast and apoplast inhibitor, respectively.

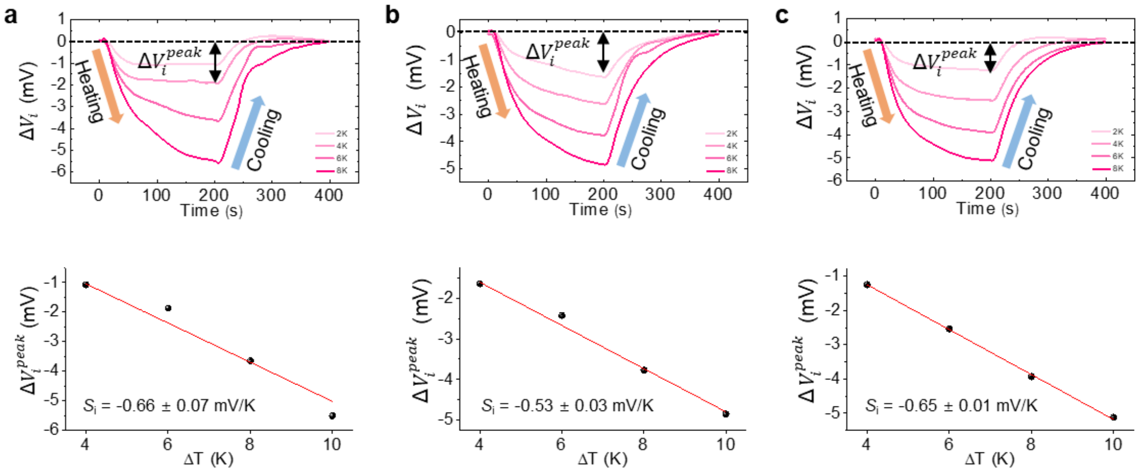

Figure S20. Effect of symplast inhibition on ionic thermovoltage. Measured ionic thermovoltage (ΔV_{i}, mV) curves and corresponding $\Delta \boldsymbol{V}_{\boldsymbol{i}}^{\boldsymbol{peak}}$ plots as a function of time for fresh leaves treated with a symplast inhibitor solution for: a) 1 day, b) 2 days, and c) 3 days. $\Delta \boldsymbol{V}_{\boldsymbol{i}}^{\boldsymbol{peak}}$ represents the peak ΔV_{i} observed upon heating at each temperature difference (ΔT).

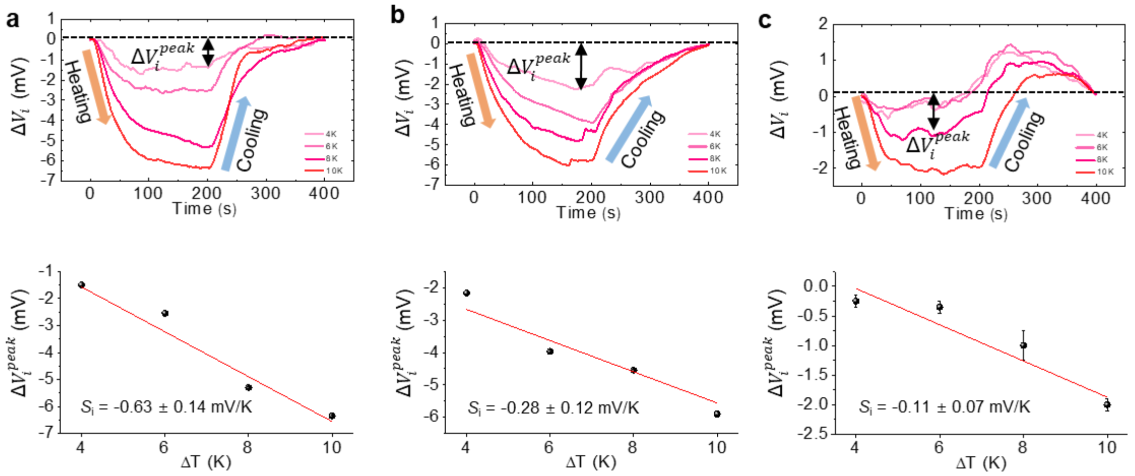

Figure S21. Effect of apoplast inhibition on ionic thermovoltage. Measured ionic thermovoltage (ΔV_i, mV) curves and corresponding $\boldsymbol{\Delta V_i^{peak}}$ plots as a function of time for fresh leaves treated with an apoplast inhibitor solution for: a) 1 day, b) 2 days, and c) 3 days. $\boldsymbol{\Delta V_i^{peak}}$ represents the peak ΔV_i observed upon heating at each temperature difference (ΔT).

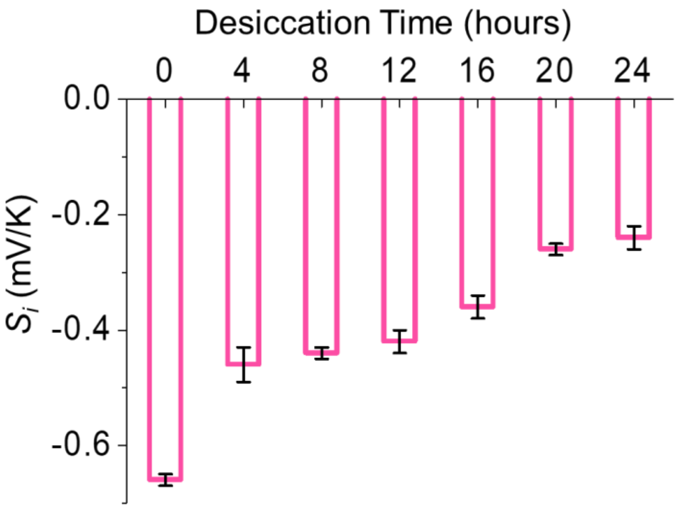

Figure S22. Effect of sealing on thermopower. Plot of ionic Seebeck coefficient (S_i, mV/K) of leaves sealed with Scotch tape as a function of desiccation time (hours).

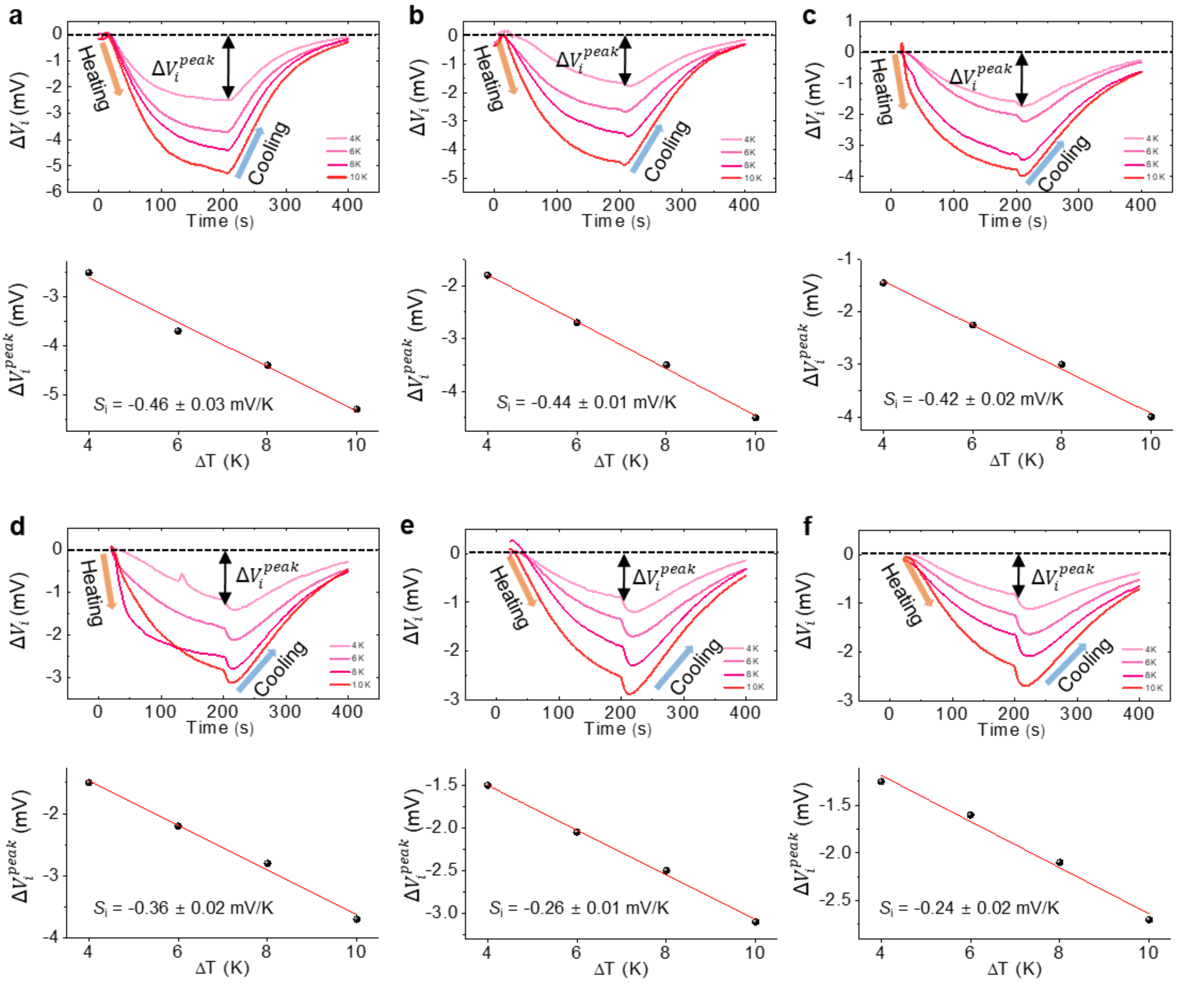

Figure S23. Effect of sealing on thermopower. Measured ionic thermovoltage (ΔV_i, mV) curves and corresponding $\boldsymbol{\Delta V_i^{peak}}$ plots as a function of time for leaves desiccated for: a) 4, b) 8, c) 12, d) 16, e) 20, and f) 24 hours. $\boldsymbol{\Delta V_i^{peak}}$ represents the peak thermovoltage observed upon heating at each temperature difference (ΔT).

a

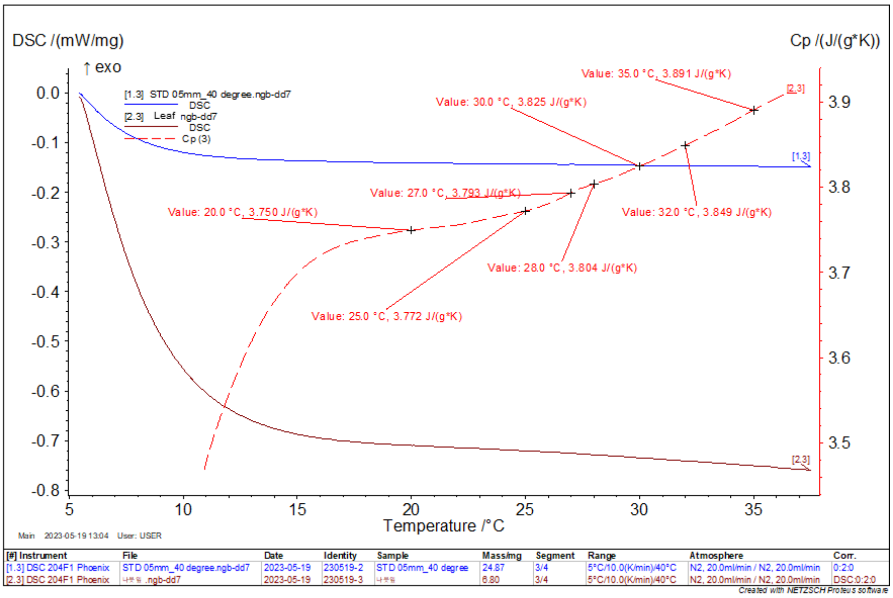

b

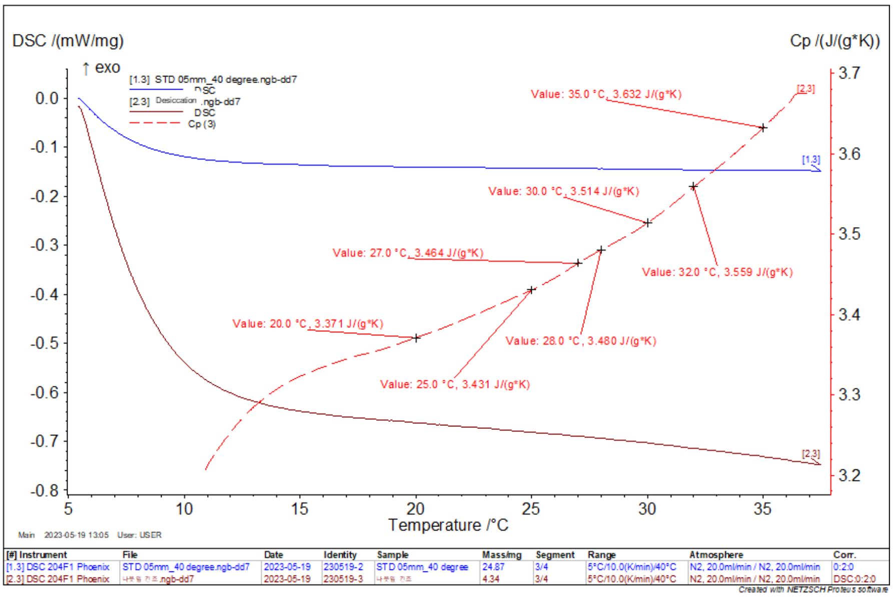

Figure S24. Heat capacity (C_p) for a) fresh and b) 24-hour desiccated leaf samples, measured by differential scanning calorimeter (DSC).

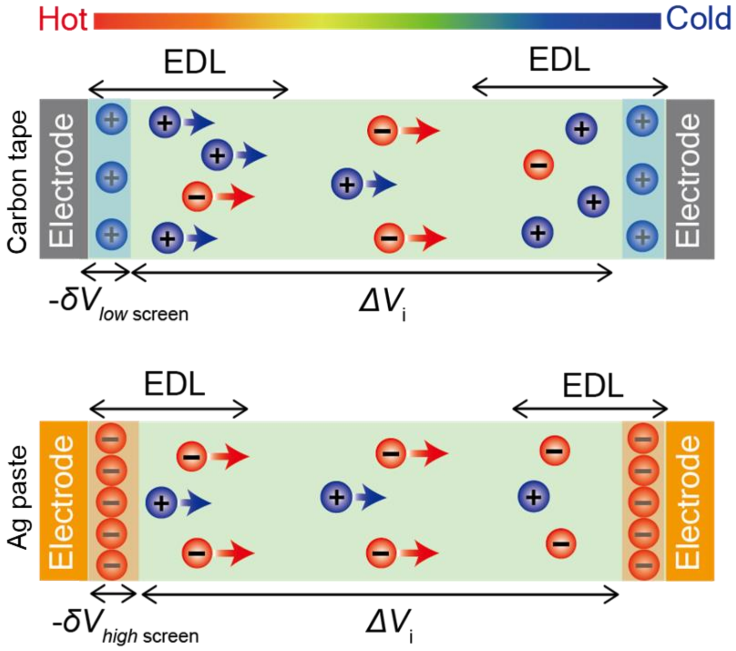

Figure S25. Effect of electrode type on thermodiffusion behavior. Schematic illustration showing the effect of an electrode type (carbon tape vs. Ag paste) on ΔV_i. Both carbon tape and Ag paste electrodes form an electrical double layer (EDL) at the electrode–electrolyte interface, comprising a compact Stern layer and a surrounding diffuse layer. Notably, the Stern layer contributes to the generation of a screening potential ($-\delta V_{screen}$) that partially offsets the measured thermovoltage.

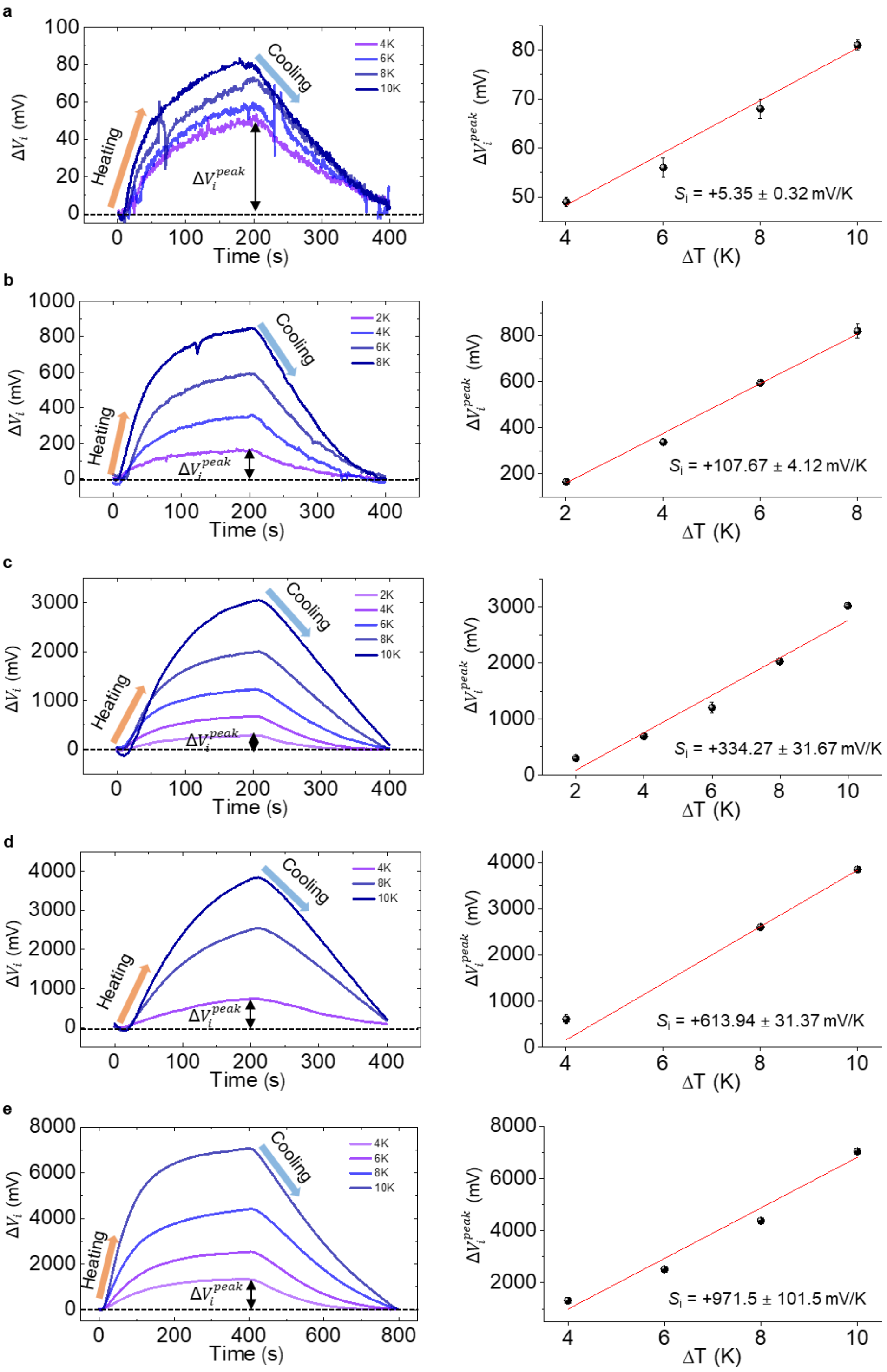

Figure S26. Effect of desiccation time on thermopower with carbon tape electrodes. Measured ionic thermovoltage (ΔV_i, mV) curves and corresponding $\Delta \boldsymbol{V_i^{peak}}$ plots as a function of desiccation time for leaves with carbon tape electrodes, desiccated for: a) fresh, b) 1 day, c) 2 days, d) 3 days, and e) 4 days. $\Delta \boldsymbol{V_i^{peak}}$ represents the peak thermovoltage observed upon heating at each temperature difference (ΔT).

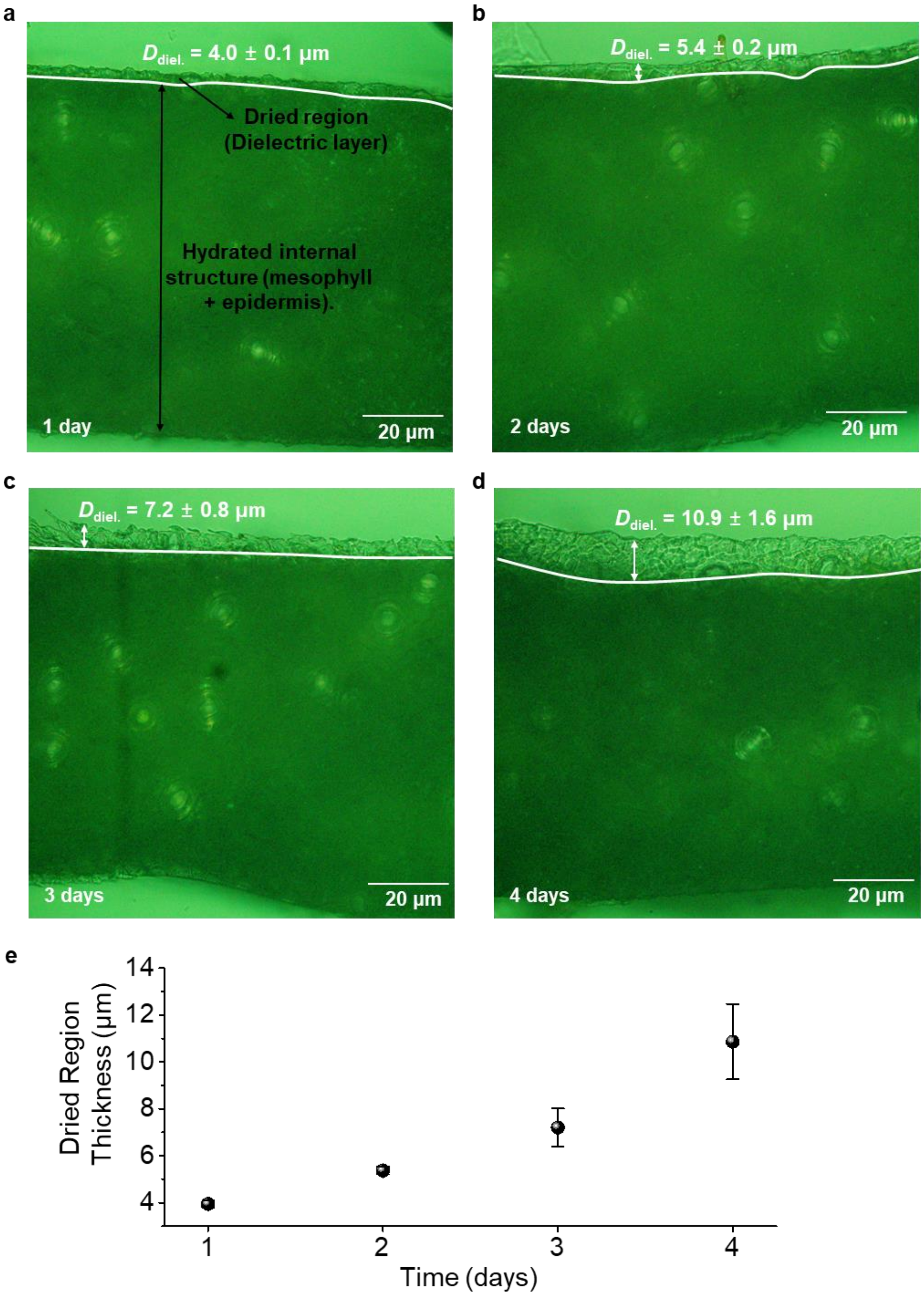

Figure S27. Optical microscopy cross-sectional images showing the progression of the dried region (dielectric layer) in the leaf after a) 1 day, b) 2 days, c) 3 days, and d) 4 days of desiccation. e) Plot of dried layer thickness as a function of desiccation time. Error bars indicate the standard deviation obtained from five independent measurements for each data point.

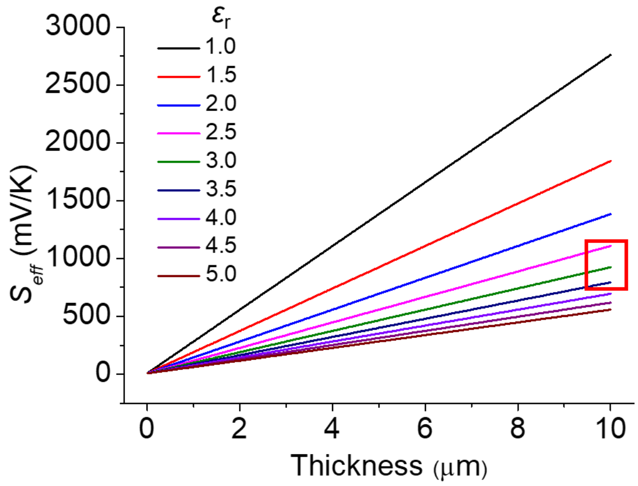

Figure S28. Effect of the relative permittivity ε_r on simulated $S_{eff.}$. The variation of the simulated S_{eff} value according to the relative permittivity ε_r from 1 to 5. As highlighted by the red box, the simulated Seebeck coefficient ($S_{eff.}$) closely matches the experimental value of 971 mV/K when the ε_r is in the range of 2.5 to 3.5.

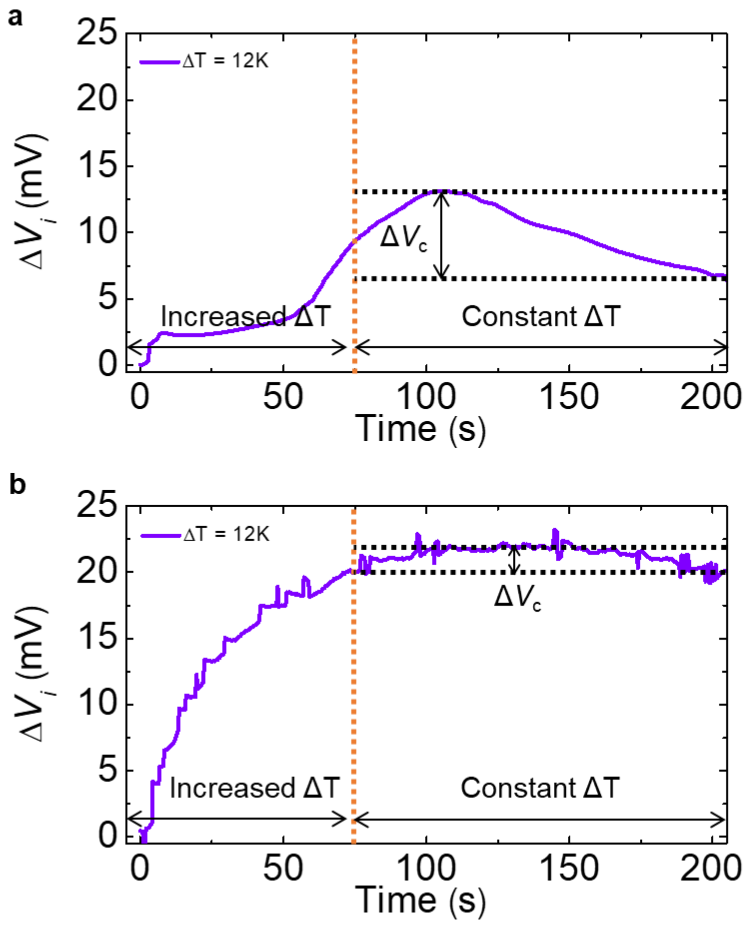

Figure S29. Effect of the dielectric layer on ΔV_{i} stability. Measured ionic thermovoltage (ΔV_{i}, mV) curves for cellulose paper soaked in 1 M NaOH solution with: a) a bare Cu electrode, and b) a Cu electrode coated with a polydimethylsiloxane (PDMS) layer (~10 µm thickness). The ΔV_{c} value represents the variation in ΔV_{i} measured over a constant ΔT range. For bare Cu electrodes, ΔV_{c} is approximately 7 mV—comparable to ΔV_{i} during heating—indicating significant signal instability. In contrast, PDMS-coated electrodes show $\Delta V_{\mathrm{c}} \approx 2$ mV, confirming improved stability at constant ΔT.

a

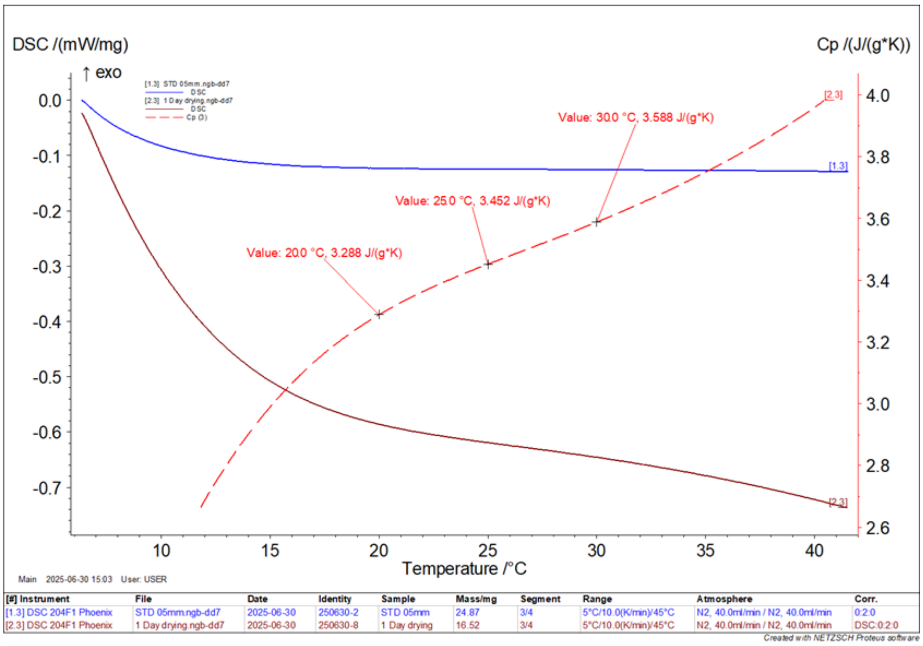

b

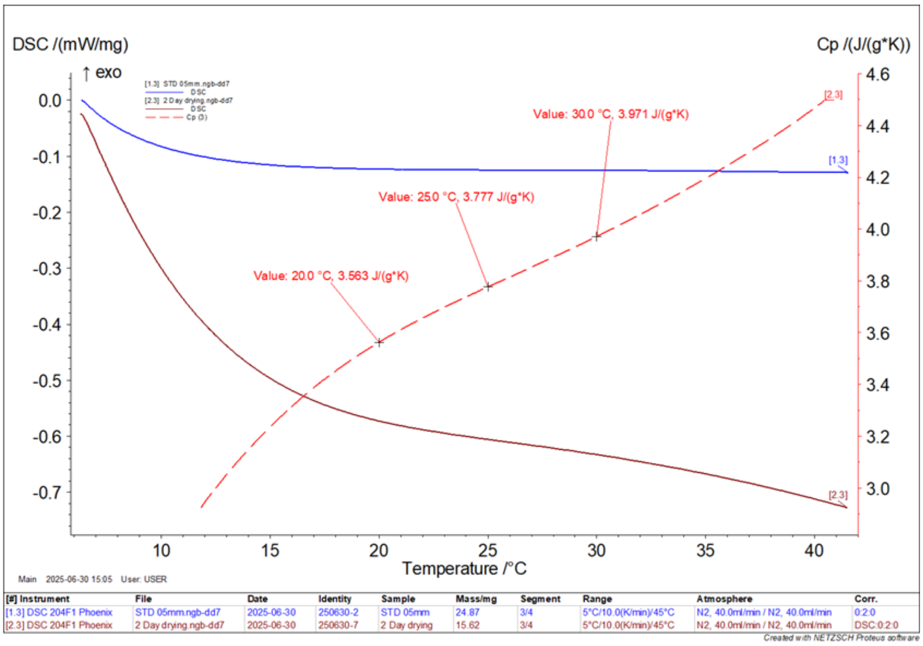

Figure S30. Heat capacity (C_p) for a) 1-day and b) 2-day desiccated leaf samples with carbon tape electrodes, measured by differential scanning calorimeter (DSC).

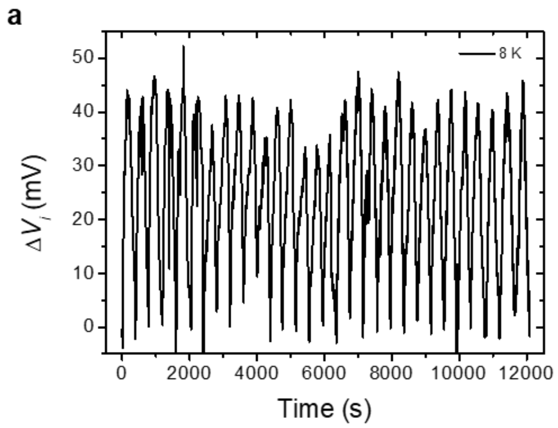

Figure S31. a) Long-term ΔV_i response recorded over 30 cycles of light on (heating) and off (cooling) in the intact leaf-based TD cell. b) Measured ΔV_i for each cycle. The red line represents the average value of the measured ΔV_i.

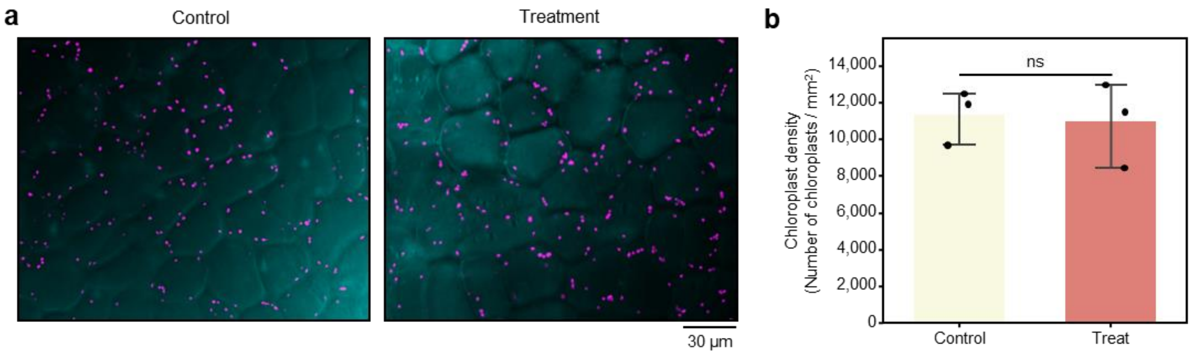

Figure S32. a) Confocal images of the upper epidermis of a control fresh leaf (left) and a leaf treated with electrode/photothermal stimulation (right). Chloroplast autofluorescence is shown in magenta; cell walls stained with Calcofluor White appear in sky blue. Scale bar = 30 µm. b) Quantification of chloroplast density on the upper surface. Chloroplasts were counted in three randomly selected 275 × 232 μm^2 fields and expressed as chloroplasts per mm^2. Data are presented as mean, and error bars represent 95% confidence intervals. Statistical significance was assessed by Student's t-test (ns, not significant).

Supporting Tables

Table S1. Ion species and concentration. Ion concentration (ppm) in the cold and hot sections of fresh and 24-hour desiccated leaves, measured using ion chromatography.

Condition	Type	Ion species	Cold (ppm)	Hot (ppm)
Fresh	Anion	NO_3^-	116	132
		Cl^-	34.1	34.4
		PO_4^{3-}	42.3	38.0
	Cation	K^+	165	178
		Ca^{2+}	16.7	16.1
		Mg^{2+}	12.1	11.3
24 h Desiccation	Anion	NO_3^-	136	169
		Cl^-	29.1	31.3
		PO_4^{3-}	40.2	35.5
	Cation	K^+	332	335
		Ca^{2+}	27.4	37.4
		Mg^{2+}	9.6	11.3

Table S2. Parameter values used in simulation.

Parameter	Value	Description
ε_r	1.0 – 5.0	Cellulose dielectric range
ε_0	8.85×10^{-12} F/m	Vacuum permittivity
σ	2.44×10^{-5} C/m^2	Optimized hot side surface charge
d	0 to 10^{-5} m	Dielectric thickness
ΔT	10 K	Temperature gradient
$V_{electrolyte}$	80.0 mV	Electrolyte thermodiffusion contribution

Table S3. S_i and *ZT* values reported in the literature.

Entry	Matrix	S_i (mV/K)	*ZT*	Ref.
**1**	Polyelectrolytes	7.2	0.00011	Bonetti et al. (*47*)
**2**		7.9	0.44	Kim et al. (*45*)
**3**		1.1	0.016	Chang et al. (*46*)
**4**		4.0	0.012	Wang et al. (*43*)
**5**		9.0	0.0032	Kim et al. (*41*)
**6**	PEO[a]	11.1	0.00135	Zhao et al. (*44*)
**7**	Cellulose/PEO	24.0	0.71	Li et al. (*8*)
**8**	PVDF-HFP[b]	26.1	0.75	Cheng et al. (*29*)
**9**	WPU[c]	34.5	1.3	Fang et al. (*38*)
**10**	SiO_2	14.8	1.47	He et al. (*37*)
**11**	PVDF-HFP	25.4	1.78	Liu et al. (*48*)
**12**	BC[d]	18.0	1.33	Liu et al. (*7*)
**13**	PU[e]	25.6	0.99	Xu et al. (*36*)
**14**	WPU	19.5	0.48	Zhao et al. (*52*)
**15**	PANI/PAAMPSA[f]	8.1	1.04	Akbar et al. (*39*)
**16**	Graphene oxide	12.6	0.85	Jeong et al. (*28*)
**17**	PVA[g]	42.8	5.18	He et al. (*34*)
**18**	PMMA[h]/SWCNT	4.2	0.087	Duan et al. (*42*)
**19**	Lignin	13	3.75	Muddasar et al. (*50*)
**20**	Graphene oxide	76.7	0.19	Sun et al. (*49*)
**21**	PVDF-HFP	43.8	6.1	Liu et al. (*51*)

[a]Poly(ethylene oxide); [b]Poly(vinylidene fluoride-co-hexafluoropropylene); [c]Aqueous polyurethane; [d]Bacterial cellulose; [e]Polyurethane; [f]Polyaniline-poly(2-acrylamido-2-methyl-1-propanesulfonic acid); [g]Polyvinyl alcohol; [h]Poly(methyl methacrylate).

Table S4. Summary of thermal power (*S*), ion conductivity (σ) power factor (*PF*), thermal conductivity (*K*), and figure of merit (*ZT*) of leaves with carbon tape electrodes depending on the desiccation conditions.

Condition	*S* (mVK^{-1})	σ (mS/cm)	*PF* ($\mu Wm^{-1}K^{-2}$)	*K* ($Wm^{-1}K^{-1}$)	*ZT* at RT
**1-day desiccation**	107.67	0.82	946.88	0.32	0.87
**2-day desiccation**	334.27	0.64	7091.41	0.38	5.62
